# The dietary sweetener sucralose is a negative modulator of T cell-mediated responses

**DOI:** 10.1038/s41586-023-05801-6

**Published:** 2023-03-15

**Authors:** Fabio Zani, Julianna Blagih, Tim Gruber, Michael D. Buck, Nicholas Jones, Marc Hennequart, Clare L. Newell, Steven E. Pilley, Pablo Soro-Barrio, Gavin Kelly, Nathalie M. Legrave, Eric C. Cheung, Ian S. Gilmore, Alex P. Gould, Cristina Garcia-Caceres, Karen H. Vousden

**Affiliations:** 1 p53 and Metabolism Laboratory, The Francis Crick Institute, London, UK; 2 University of Montreal, Maisonneuve-Rosemont Hospital Research Centre, Montreal, Quebec Canada; 3 Institute for Diabetes and Obesity, Helmholtz Diabetes Center, Helmholtz Zentrum München and German Center for Diabetes Research (DZD), Neuherberg, Germany; 4 Immunobiology Laboratory, The Francis Crick Institute, London, UK; 5 Institute of Life Science, Swansea University Medical School, Swansea University, Swansea, UK; 6 National Physical Laboratory, Teddington, UK; 7 Laboratory of Physiology and Metabolism, The Francis Crick Institute, London, UK; 8 Bioinformatics and Biostatistics Science Technology Platform, The Francis Crick Institute, London, UK; 9 Metabolomics Science Technology Platform, The Francis Crick Institute, London, UK; 10 Medizinische Klinik und Poliklinik IV, Klinikum der Universität, Ludwig-Maximilians-Universität München, Munich, Germany

**Keywords:** Adaptive immunity, Cancer microenvironment

## Abstract

Artificial sweeteners are used as calorie-free sugar substitutes in many food products and their consumption has increased substantially over the past years^1^. Although generally regarded as safe, some concerns have been raised about the long-term safety of the consumption of certain sweeteners^2–5^. In this study, we show that the intake of high doses of sucralose in mice results in immunomodulatory effects by limiting T cell proliferation and T cell differentiation. Mechanistically, sucralose affects the membrane order of T cells, accompanied by a reduced efficiency of T cell receptor signalling and intracellular calcium mobilization. Mice given sucralose show decreased CD8^+^ T cell antigen-specific responses in subcutaneous cancer models and bacterial infection models, and reduced T cell function in models of T cell-mediated autoimmunity. Overall, these findings suggest that a high intake of sucralose can dampen T cell-mediated responses, an effect that could be used in therapy to mitigate T cell-dependent autoimmune disorders.

## Abstract (web summary)

Consumption of high doses of the sweetener sucralose has immunomodulatory effects in mice, as a result of reduced T cell proliferation and differentiation.

## Main

**Fig. 1:**
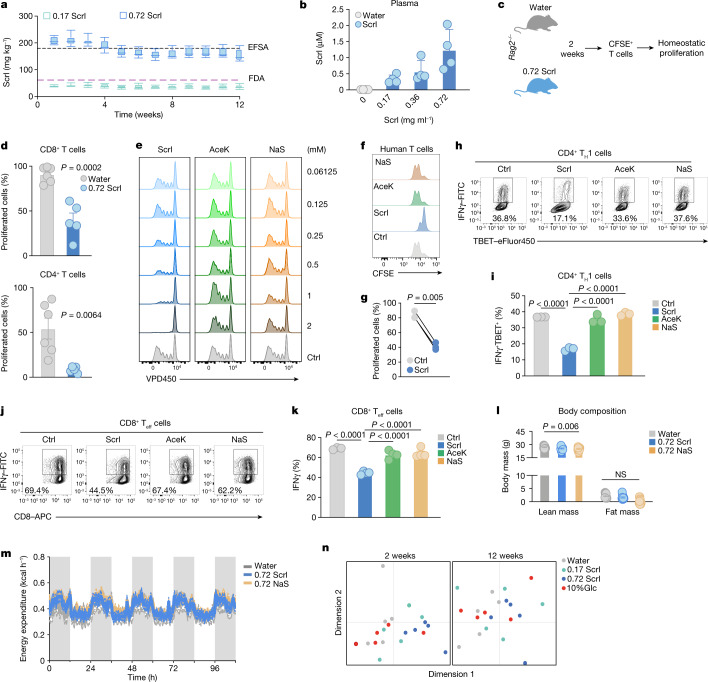
Sucralose impairs T cell proliferation and differentiation. **a**, Sucralose (Scrl) intake in mice given 0.72 mg ml^−1^ (blue; *n* = 6) or 0.17 mg ml^−1^ (aquamarine; *n* = 6) Scrl. In box plots, whiskers show the minimum and maximum values, box margins represent the first and third quartile and the central line is the median value. Dashed lines indicate the BSA-adjusted EFSA (black) and FDA (purple) maximum ADI. Scrl concentrations are indicated in mg ml^−1^ throughout. **b**, Circulating Scrl levels in mice given water containing different Scrl concentrations for 2 weeks. *n* = 4 individual mice per condition. **c**, Schematic of the experimental design. CFSE, carboxyfluorescein succinimidyl ester. **d**, Homeostatic proliferation of CD8^+^ and CD4^+^ donor T cells in individual *Rag2*^−/−^ recipient mice given plain water (*n* = 6) or Scrl (*n* = 5). **e**, Histograms of CD8^+^ T cell proliferation in the presence of Scrl, AceK, NaS or control medium (Ctrl). **f**, Human CD8^+^ T cell proliferation in the presence of Scrl, AceK, NaS or control medium. **g**, Paired comparison of the percentage of proliferated CD8^+^ T cells in **f**. *n* = 3 independent donors. **h**, Representative flow cytometry plot of in vitro polarized CD4^+^ T_H_1 cells expressing IFNγ and TBET (also known as TBX21). **i**, The percentage of T_H_1 cells in **h**. *n* = 3 technical replicates per condition. **j**, Representative flow cytometry plot of CD8^+^ T cells expressing CD8 and IFNγ. **k**, Quantification of CD8^+^IFNγ^+^ cells in **j**. *n* = 3 (Ctrl) or *n* = 4 (Scrl, AceK and NaS) technical replicates per condition. **l**,**m**, Mice were given plain water (*n* = 9) or 0.72 mg ml^−1^ of either Scrl (*n* = 12) or NaS (*n* = 11). **l**, Body composition (lean versus fat mass). **m**, Average energy expenditure measured continuously during night (grey area) and day (white area). **n**, Multidimensional scaling of the faecal gut microbiome from mice given water (*n* = 5), 0.72 mg ml^−1^ Scrl (*n* = 5), 0.17 mg ml^−1^ Scrl (*n* = 5) or 10% (w/v) glucose (*n* = 5) for 2 (left) or 12 (right) weeks. Data are mean ± s.d. (**b**,**i**,**k**) or mean ± s.e.m. (**d**,**l**,**m**). Significance was tested using unpaired (**d**) or paired (**g**) two-tailed Student’s *t*-test; one-way ANOVA with Tukey’s (**i**,**k**) or Dunnet’s multiple comparison test for lean and fat mass independently (**l**) or two-way ANOVA (**m**). Data are representative of two (**d**) or three (**e**,**h**–**k**) independent experiments. Source data

**Extended Data Fig. 1:**
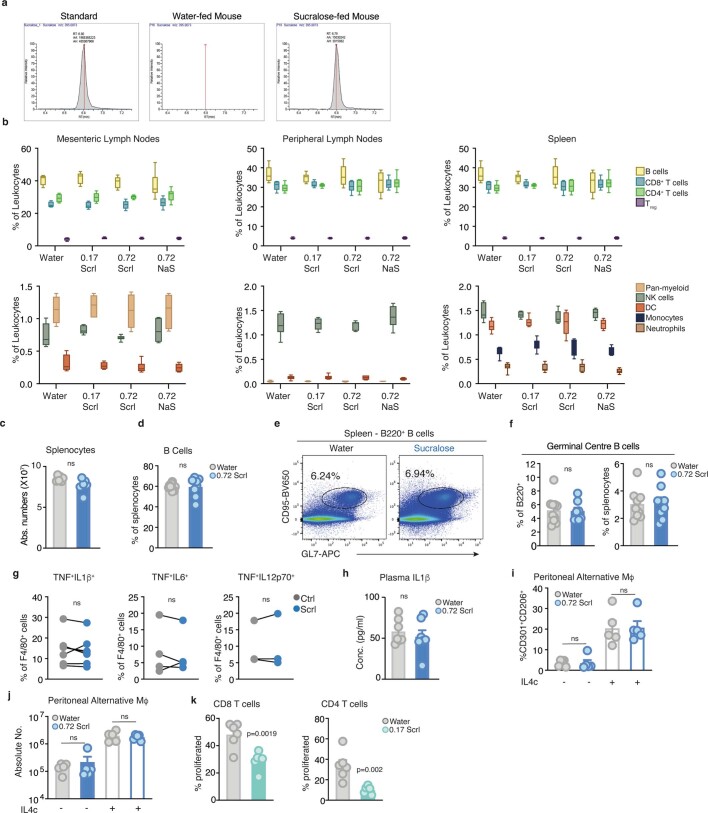
Sucralose detection in mice and its effect on macrophage, B cell and T cell responses. **a**). Sucralose peak detection by LC-MS in the plasma of mice fed with water or 0.72 mg ml^−1^ of sucralose for 2 weeks. **b**) Box plots of frequencies of B220^+^ B cells, CD8^+^ T cells, CD4^+^ T cells, CD4^+^FOXP3^+^ T cells (T_reg_), CD11b^+^ pan myeloid population, CD11b^+^NK1.1^+^ cells (NK cells), CD11c^+^ dendritic cells (DCs), monocytes (CD11b^+^Ly6C^+^) and neutrophils (CD11b^+^Ly6G^+^) within mesenteric lymph nodes, peripheral lymph nodes, and the spleen of mice fed with water), 0.17 mgml^−1^ Scrl, 0.72 mg ml^−1^ Scrl, and 0.72 mg ml^−1^ sodium saccharin (NaS) (n = 6 per treatment) for over 12 weeks. As assessed by flow cytometry. Min-max box plot: centre lines show median, box limits are 1^st^ and 3^rd^ quartiles, with whiskers indicating the min and max value. **(c-f)** C57BL/6J mice fed with water (n = 10) or 0.72 mg ml^−1^ Scrl (n = 8) and immunized with sheep red blood cells (sRBC). Each dot represents a biological replicate. **c)** Total splenocyte numbers at day 7 post sRBC immunization. **d**) Percentage of splenic B220^+^ B cells 7 days post SRBC immunization. **e**) Representative density plot of gated B220^+^ B cells and the frequency of germinal centre (GC) B cells (GL7^+^CD95^+^) at day 7 post immunization. **f**) Quantification of the percentage of GC B cells as depicted in Extended Data Fig. 1e, of the splenic B220^+^ B cell population (left) and as a percentage of total splenocytes (right). **g)** Pairwise comparison of pro-IL1β^+^TNF^+^ (n = 7/condition), TNF^+^IL6^+^ (n = 4/condition) and TNF^+^IL12p70^+^ (n = 3/condition) production assessed by flow cytometry in bone marrow derived macrophages (BMDMs) stimulated with lipopolysaccharide (LPS) either in control media or in presence of 0.5 mM of Scrl. Paired dots indicate biologically independent samples. **h**) IL1β plasma concentration from water (n = 7) or 0.72 mg ml^−1^ sucralose (n = 7) fed C57BL/6J mice for 2 weeks prior to LPS challenge (0.1 mg kg^−1^). (**i–j**) Generation of alternatively activated macrophages (CD11b^+^F4/80^+^CD301^+^CD206^+^) in C57BL/6J mice intraperitoneally injected with IL4 complex (IL4c) or PBS fed water (or 0.72 mg ml^−1^ Scrl . Each dot represents a single mouse. N = 5 biological replicates per group. **i**) Frequencies. **j**) Absolute numbers. **k**) Homeostatic proliferation at day 3 of naïve CFSE-loaded CD8^+^ and CD4^+^ T cells injected to *Rag2*^*−/−*^ recipients fed water (n = 6) or 0.17 mg ml^−1^ Scrl (n = 5) for two weeks and until the end of the experiment. Means represent ± s.e.m for biological replicates (**c**, **d, f**, **g**, **h**, **i**, **j**, **k**). Statistical significance was determined using 2-tailed unpaired (**c**, **d**, **f, h, k**) or paired (**g**) Student’s t test; one-way ANOVA with Tukey’s multiple comparison test (**i, j**), or ordinary one-way ANOVA for identical immune populations/condition (**b**). Data are representative of 2 (**h–i**) or 3 (**c–f, k**) independent experiments. Source data

Sucralose is a commonly used, calorie-free sweetener that is about 600 times sweeter than sucrose^6^. Despite its limited absorption^7^, circulating sucralose can be detected in humans following consumption of sucralose-containing food or drinks^8^, with consumption of 250 mg sucralose resulting in plasma sucralose levels of around 1 μM within 90–120 min (ref. ^8^). The maximum acceptable daily intake (ADI) of sucralose for humans has been established as 15 mg per kg (body weight) by the European Food Safety Authority (EFSA) or 5 mg per kg (body weight) by the US Food and Drug Administration (FDA). Allometric scaling on the basis of body surface area (BSA) equivalents can be used to convert human doses of drugs to mouse doses by adjusting for the increased metabolic rate in mice^9^. By allowing mice ad libitum access to water containing 0.72 mg ml^−1^ or 0.17 mg ml^−1^ of sucralose, we calculated—using BSA equivalents—that the consumption of sucralose over 10 weeks was near the equivalent of the ADI recommended by either EFSA (at the 0.72 mg ml^−1^ dose) or FDA (at the 0.17 mg ml^−1^ dose) (Fig. 1a). As expected, we were able to detect increasing amounts of circulating sucralose corresponding with increased consumption in mice (Fig. 1b and Extended Data Fig. 1a), reaching a plasma concentration of around 1 µM at the highest dose of sucralose, consistent with the levels that can be achieved in humans^8^.

## Effect on T cell proliferation and differentiation

**Extended Data Fig. 2:**
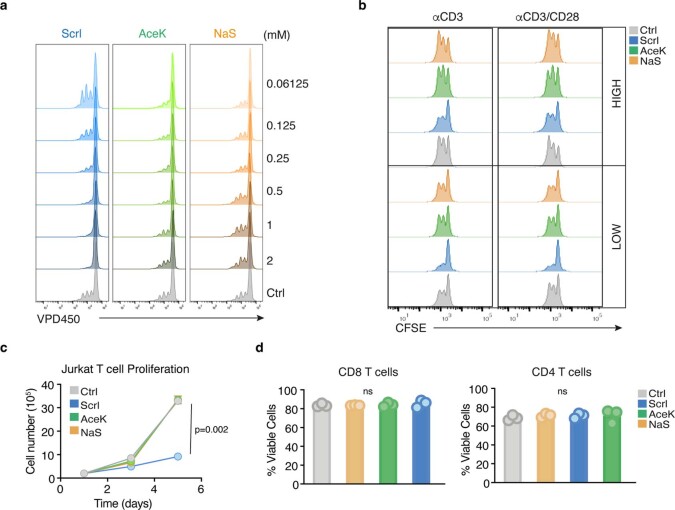
Sucralose limits T cell proliferation without affecting viability. **a**) CD4^+^ T cell proliferation assay performed with αCD3 (5 μg ml^−1^) and αCD28 (1 μg ml^−1^) for 3 days in the presence of serial dilutions of the indicated sweeteners. **b**) Proliferation of CFSE stained CD8^+^ T cells exposed to 0.5 mM of the indicated sweeteners and activated with a high dose of αCD3 (5 μg ml^−1^) or low dose of αCD3 (1 μg ml^−1^) in the presence or absence of αCD28 (1 μg ml^−1^). **c**) Jurkat T cell proliferation in control media or media supplemented with the indicated sweeteners. N = 3. **d**) Percentage of viable CD8^+^ (left) and CD4^+^ (right) T cells 24 h post activation with αCD3 (2 μg ml^−1^) and αCD28 (1 μg ml^−1^). Cell viability was determined by Fixable Viability Dye eFluor780® exclusion. Means represent ± s.e.m. for biological (**d**) or technical (**c**) replicates. Statistical significance was determined using one-way ANOVA with Tukey’s (**c**) or Dunnet’s (**d**) multiple comparison test. Data are representative of 3 independent experiments (**c**). Source data

Previous reports using different models have suggested that high doses of sucralose can have either pro-inflammatory or anti-inflammatory activities^2–4^. To test a possible effect of sucralose on the immune system, we profiled various immune compartments in mice given 0.17 or 0.72 mg ml^−1^ sucralose or the chemically unrelated sweetener sodium saccharin (NaS). In these studies, neither dose of sucralose or NaS had any detectable effect on the homeostatic levels of CD11b^+^ myeloid cells (including monocytes and neutrophils), B220^+^ B cells, CD8^+^ T cells and CD4^+^ T cells (including T regulatory (T_reg_) cells), natural killer cells and dendritic cells (Extended Data Fig. 1b). To assess the effect of sucralose on immune responses, we challenged mice given 0.72 mg ml^−1^ sucralose or water with sheep red blood cells (sRBCs) to activate a germinal centre B cell response or with lipopolysaccharide (LPS) or interleukin-4 (IL-4) complex to activate a myeloid response. Sucralose did not change the number of splenocytes or B220^+^ B cells or affect germinal centre B cell formation (Extended Data Fig. 1c–f). Similarly, bone marrow-derived macrophages cultured in sucralose containing media did not display altered IL-1β, IL-6 or IL-12p70 production upon LPS stimulation, nor were there differences in LPS-induced IL-1β production in plasma of mice given sucralose compared with controls (Extended Data Fig. 1g,h). Finally, we did not observe any effect on the alternative activation or expansion of macrophages elicited by IL-4 complex in mice exposed to sucralose (Extended Data Fig. 1i,j). To examine the effect of sucralose on T cell proliferation, we measured the homeostatic expansion of donor T cells in sucralose-treated *Rag2*^−/−^ recipient mice (Fig. 1c). Both CD8^+^ and CD4^+^ T cells showed reduced homeostatic proliferation in sucralose-treated mice at both doses (Fig. 1d and Extended Data Fig. 1k). To evaluate whether sucralose has a direct effect on T cells, we performed in vitro T cell proliferation assays in the presence of increasing doses of sucralose or two chemically unrelated sweeteners: acesulfame potassium (AceK) and NaS. Sucralose alone showed a dose-dependent ability to inhibit the proliferation of CD8^+^ and CD4^+^ T cells (Fig. 1e and Extended Data Fig. 2a). This negative effect on T cell proliferation was observed following both high- and low-dose activation with anti-CD3 antibody, even in the presence of co-stimulation (combined anti-CD3 and anti-CD28) (anti-CD3/CD28) (Extended Data Fig. 2b). Similarly, sucralose, but not NaS or AceK, inhibited the proliferation of human CD8^+^ T cells and the human T cell leukaemia cell line, Jurkat (Fig. 1f,g and Extended Data Fig. 2c). However, none of the sweeteners affected CD8^+^ or CD4^+^ T cell viability (Extended Data Fig. 2d). T cell differentiation and effector function also have a critical role in determining T cell responses. We found that the polarization of CD4^+^ and CD8^+^ T cells towards interferon-γ (IFNγ)-producing lineages—CD4^+^ T helper 1 (T_H_1) cells and CD8^+^ effector T (T_eff_) cells, respectively—was significantly decreased in the presence of sucralose but not with the other sweeteners (Fig. 1h–k). Together, these results indicate that high sucralose exposure decreases T cell proliferation and differentiation.

## Systemic effects of sucralose

**Extended Data Fig. 3:**
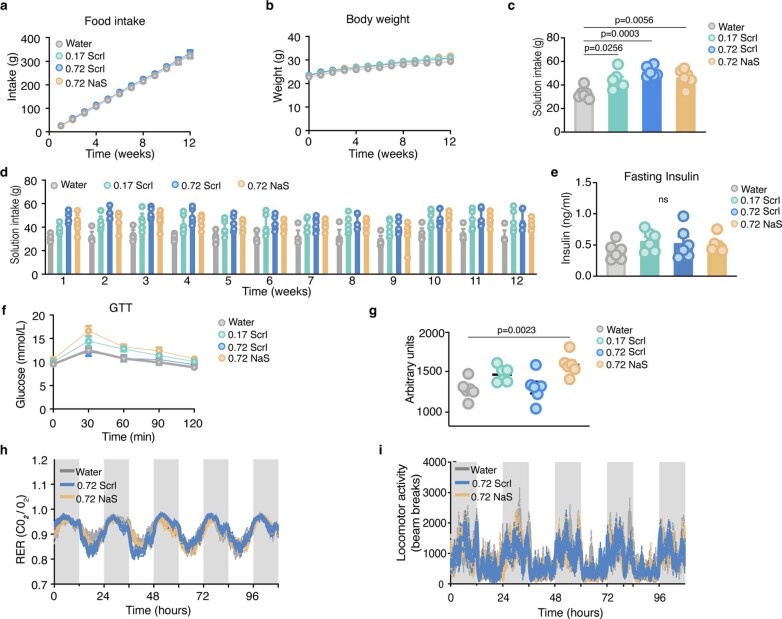
Sucralose does not affect whole body mouse metabolism. (**a–g**) Individually caged C57BL/6J mice fed water, 0.17 mg ml^−1^ Scrl, 0.72 mg ml^−1^ Scrl) or 0.72 mg ml^−1^ sodium saccharin (NaS) for 12 weeks. N = 6 per treatment. **a**) Average cumulative food intake. **b**) Average body weight. **c**) Average solution intake. **d**) Weekly solution intake. **e**) Fasting plasma insulin levels. (**f–g**) Glucose tolerance test (GTT) of mice receiving an oral bolus of 2 mg kg^−1^ glucose. **f**) Blood glucose. **g**) Area under the curve calculated using the trapezoid method for the GTT. **h–i**) C57BL/6J mice fed for over 8 weeks with water (n = 8 for RER and 9 for locomotor activity), 0.72 mg ml^−1^ Scrl (n = 12) or 0.72 mg ml^−1^ NaS (n = 11). **h**) Respiratory Exchange Ratio (RER). **i**) Locomotor activity. Data are displayed as mean ± s.d. (**d**) ± s.e.m. (**a**, **b**, **c**, **e**, **f**, **g**, **h**, **i**). Each dot represents a single mouse. Statistical significance was determined using one-way Anova with Tukey’s (**a**, **b**, **c**) or Dunnet’s (**e**, **g**) multiple comparison test. Source data

**Extended Data Fig. 4:**
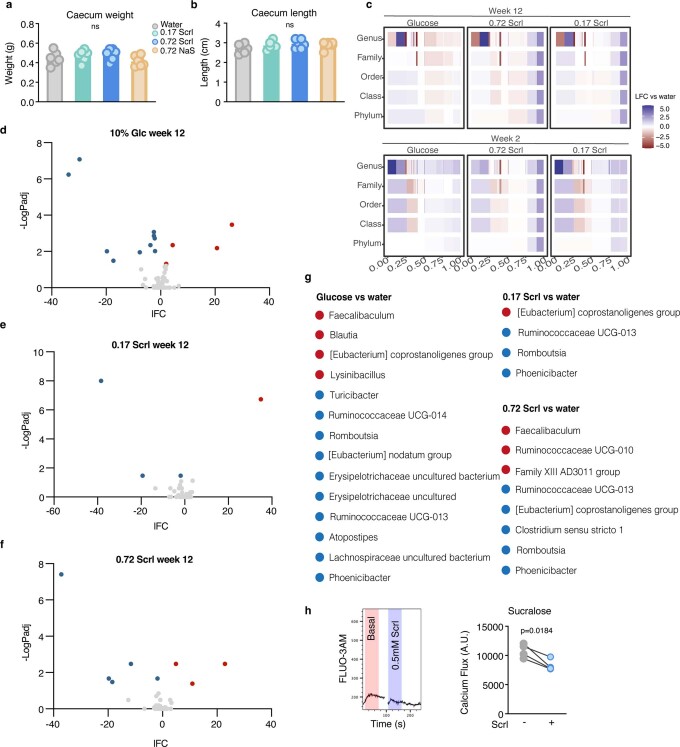
Microbiome analysis of mice treated with sweeteners and sucralose effect on calcium flux. (**a–b**) C57BL/6J mice fed water, 0.17 mg ml^−1^ Scrl, 0.72 mg ml^−1^ Scrl or 0.72 mg ml^−1^ NaS for 12 weeks. Each dot represents an individual mouse. N = 6 per treatment. **a**) Caecum weight. **b**) Caecum length. (**c–g**) Gut microbiome analysis of mice fed for 2 and 12 weeks with water), 0.17 mg ml^−1^ Scrl, 0.72 mg ml^−1^ Scrl or 10% w/v glucose. N = 5 per treatment. **c**) Heatmaps showing alteration in abundance data in mice exposed to different drinking solutions as indicated compared to time-matched samples obtained from water-fed mice. Top graphs: samples collected after 12 weeks exposure; lower graphs samples collected after 2 weeks exposure. The variation of abundance is shown at Phylum, Class, Order, Family and Genus level. (**d–f**) Volcano plots of regulated genera in samples collected after 12 weeks of treatment. Water-treated animals were used as control. The fold changes were estimated using a negative binomial model from the DESeq2 package in R using its default settings for accounting for different library sizes between samples. A generalized linear model accounted for batch along with the interaction of treatment and time, to provide estimates of time effects within treatment (and vice versa). P-values were calculated using a Wald test and then adjusted using the Benjamini-Hochberg method to control for false discovery rate. Comparisons: **d**) 10% glucose vs water, **e**) 0.17 mg ml^−1^ Scrl vs water and **f**) 0.72 mg ml^−1^ Scrl vs water. Genera with a logarithmic fold change > 0.6 and an adjusted P-value < 0.05 are considered upregulated; Genera with a logarithmic fold change < 0.6 and an adjusted P-value < 0.05 are considered downregulated. **g**) List of upregulated (red) or downregulated (blue) genera in the different comparison as indicated in (**d**, **e**, **f**). **h**) Representative kinetics diagram (left) of FLUO-3AM calcium flux in response to 0.5 mM Scrl and corresponding quantification (right) of FLUO-3AM intensity in Jurkat T cells. N = 4 technical replicates. Data are representative of 3 independent experiments. The means represents ± s.e.m. for biological replicates (**a**, **b**), one-way ANOVA with Dunnet’s multiple comparison test (**a**, **b**), and 2-tailed paired Student’s t test (**h**) were used for statistical analysis. Source data

Dietary intake of sucralose has the potential to affect food intake and metabolic parameters in mice. However, we found that up to 12 weeks of exposure to either dose of sucralose or NaS did not affect food intake or body weight in mice (Extended Data Fig. 3a,b). As expected, the average intake of liquid was higher in mice given water containing sweetener (Extended Data Fig. 3c), and the consumption remained consistent over the 12-week period (Extended Data Fig. 3d). Sucralose did not alter the lean or fat mass of the mice (Fig. 1l) and did not significantly affect fasting insulin levels or glucose tolerance (Extended Data Fig. 3e–g). Finally, we detected no major sucralose-related effect on energy expenditure (Fig. 1m), respiratory exchange ratio or locomotor activity (Extended Data Fig. 3h,i). Sucralose has previously been shown to affect the gut microbiota in some^10,11^ but not all^12^ studies. We found no consistent shift in the bacterial species detected in the stool of sucralose-treated mice (Fig. 1n). Furthermore, the weight and length of the caecum did not change, and we did not detect signs of diarrhoea (such as pale watery stool) in sucralose-treated mice (Extended Data Fig. 4a,b). Closer analysis of the bacterial composition revealed larger changes in glucose-treated mice, but only minor differences in sucralose-treated mice, compared with mice given water (Extended Data Fig. 4c–g).

## No clear role for the sweet taste receptor

Our in vitro studies suggested that sucralose has a direct effect on T cells, modulating their proliferation and differentiation. The established function of sucralose is to activate the canonical sweet taste receptor (STR), a G-protein-coupled receptor that is responsible for the perception of sweet taste. The STR is a member of the type 1 taste receptors (T1Rs) and is a heterodimer comprising the T1R2 and T1R3 subunits^13^. Other members of this family include the umami receptor^14^ (a T1R1–T1R3 heterodimer) and possibly T1R3 homodimers^15^. In addition to the taste buds, the subunits of these receptors are expressed in several different cell types, including cells in the gastrointestinal tract^16^, pancreas^17^, brain^18^ and adipose tissue^19^, although analysis of published RNA expression data suggests that their expression in T cells is low^20^. There is also evidence that the STR can have a role in regulating innate immunity^21^. Sucralose, AceK and NaS have been reported to bind to different regions of T1R2 and T1R3 (ref. ^22^) and activate downstream signalling from the canonical STR (T1R2–T1R3) and possibly the T1R3 homodimer^22,23^. This binding results in increased intracellular calcium levels^24,25^, which we did not detect when we treated Jurkat T cells with sucralose in the absence of T cell receptor (TCR) stimulation (Extended Data Fig. 4h). Furthermore, even at high concentrations (2 mM), NaS and AceK did not evoke the same effect as sucralose on primary T cell responses (Fig. 1e and Extended Data Fig. 2a). Together, our data suggest that activation of the STR is unlikely to mediate the sucralose phenotype in T cells, leading us to explore alternative mechanisms to explain this effect.

## Sucralose impairs TCR-dependent proliferation

**Extended Data Fig. 5:**
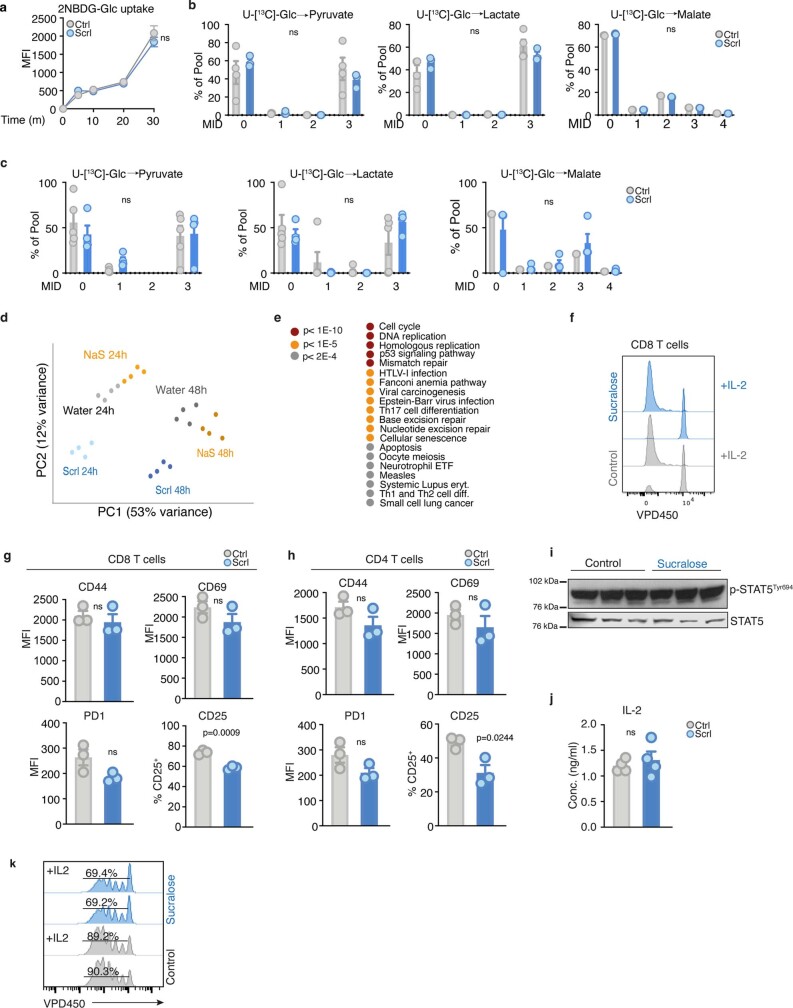
Sucralose does not impede glucose metabolism, TCR-independent proliferation, IL2-STAT5 signalling, and activation markers, but reduces membrane order. **a**) Flow cytometry analysis of 2NBDG-glucose uptake of T cells activated for 24 h with αCD3 and αCD28 in T cell media (TCM) with (Scrl) or without (Ctrl) 0.5mM sucralose. Graph shows the increase in mean fluorescence intensity (MFI) over time. N = 3 technical replicates except for sucralose at 5 min n = 2. (**b–c**) CD4^+^ (**b**) and CD8^+^ (**c**) T cells activated for 48 h in control media or in presence of 0.5 mM Scrl followed by stable isotope tracing analysis of U-[^13^C]-glucose. Mass isotopomer distribution (MID) of ^13^C_6_-glucose-derived carbon into pyruvate, lactate, and malate as indicated. N = 4 (Ctrl), n = 3 (Scrl) for CD4^+^ T cells, and n = 5 (Ctrl) and n = 4 (Scrl) for CD8^+^ T cells. Dots represent technical replicates. Values below 10^4^ were considered zero. **d**) PCA from RNAseq of CD4^+^ T cells activated with αCD3 and αCD28 for 24 h and 48 h in control medium or medium supplemented with 0.5 mM of either NaS or Scrl. Dots represent technical replicates. **e**) Top 20 enrichment pathways identified using DAVID. Pathways are ordered by p-values from most significant (top, dark red) to less significant (bottom, grey). **f**) IL2 (100 ng/ml)-induced proliferation of VPD450-stained CD8^+^ T cells in the presence or absence of 0.5 mM sucralose for 4 days. (**g–h**) Expression of the activation markers CD44, CD69, PD1 and CD25 in CD8^+^ T cells (**g**) and CD4^+^ T cells (**h**) activated for 24 h with αCD3 (2 μg ml^−1^) and αCD28 (1 μg ml^−1^) in the presence or absence of 0.5 mM Scrl. n = 3 technical replicates. **i**) Western blot analysis probing for phospho-STAT5^(Tyr694)^ and STAT5 as a loading control from protein lysates of 24 h-activated T cells in the presence of control media or 0.5 mM Scrl. Each lane is a pool of T cells collected from a single well. **j**) Concentration of IL2 detected by enzyme linked immunosorbent assay (ELISA) from supernatant of T cells activated for 24 h with αCD3/CD28. Each dot represents a single well of technical replicates (n = 4/condition). **k**) Cell proliferation histogram overlay of VPD450-loaded T cells activated with αCD3/CD28, with or without 20 μg -ml^−1^ of IL-2, and either in control medium (grey) or in presence of 0.5 mM Scrl (blue). Data presented with mean value ± s.e.m. (**a**, **b, c**, **g**, **h**) or ± s.d. (**j**) Statistical significance was tested using, mixed-effects model (REML) with Sidak’s multiple comparison test (**a**); 2-way ANOVA (**b, c**), unpaired (**g**–**j**) 2-tailed student’s t-test. Data are representative of 3 independent experiments (**a, f–k**). Source data

Sucralose could potentially affect T cell metabolism, but we found no difference in glucose uptake between sucralose and control treated cells (Extended Data Fig. 5a) and no change in glucose metabolism, as measured by the conversion of labelled [^13^C_6_]glucose into pyruvate, lactate or malate in either CD4^+^ or CD8^+^ T cells (Extended Data Fig. 5b,c). Taking an unbiased approach, we carried out RNA-sequencing analysis (RNA-seq) on activated T cells exposed to sucralose, NaS or control medium for 24 and 48 h. Principal component analysis (PCA) showed that T cells activated in the presence of sucralose displayed a unique expression profile compared with control cells or cells treated with NaS (Extended Data Fig. 5d). Enrichment analysis identified several pathways affected by sucralose, including those associated with proliferation, as expected (Extended Data Fig. 5e). As these T cells were activated through their TCR with anti-CD3, we tested whether the effect of sucralose was specific to TCR-dependent proliferation using a high concentration (100 ng ml^−1^) of IL-2 to induce TCR-independent proliferation^26^. CD8^+^ T cell expansion induced by this approach was not reduced by sucralose (Extended Data Fig. 5f), suggesting that sucralose specifically impedes TCR-dependent proliferation. Upon activation, T cells upregulate migratory receptors, cytokine receptors and costimulatory and inhibitory molecules^27^. Sucralose had no significant effect on the expression of CD44, CD69 or PD1 activation markers (Extended Data Fig. 5g,h), and despite lower CD25 expression, its downstream targets—STAT5 phosphorylation and IL-2 levels—were not modified by sucralose (Extended Data Fig. 5i,j). Furthermore, IL-2 supplementation did not rescue TCR-mediated proliferation in the presence of sucralose (Extended Data Fig. 5k). These results indicate that sucralose does not affect all aspects of T cell activation.

## Effect on cell membranes and PLCγ1 activation

**Fig. 2:**
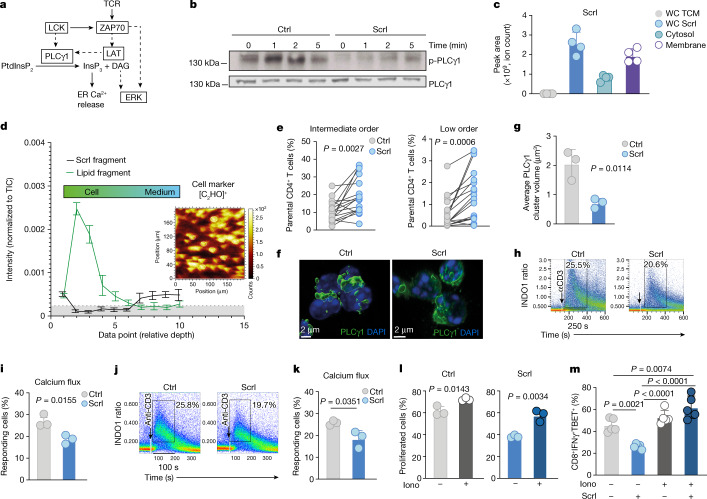
Sucralose decreases intracellular calcium flux downstream of the TCR. **a**, Schematic of the TCR signalling cascade. ER, endoplasmic reticulum. **b**, Western blot of phosphorylated and total PLCγ1 in anti-CD3-stimulated T cell lysates. **c**, Liquid chromatography–mass spectrometry (LC–MS) quantification of Scrl in whole-cell lysate, cytosolic fraction and membrane fraction of Jurkat T cells exposed to 0.5 mM Scrl. Whole-cell lysate of Jurkat T cells grown in T cell medium with (WC Scrl) or without (WC TCM) 0.5 mM Scrl are shown as controls. *n* = 4 independent preparations. **d**, Cryogenic OrbiSIMS analysis of Scrl-treated mouse T cells shows the intensity–depth profile above background (grey shaded area) for Scrl [CClNa_2_O]^+^ and lipid cell marker [C_16_H_11_0_5_]^+^ fragments. Inset, ion intensity map for the [C_2_HO]^+^ cell marker (*m*/*z* = 40.99), illustrating the 8 cells quantified (circled). TIC, total ion count. **e**, The percentage of CD4^+^ T cells with intermediate (left) and low (right) membrane order activated in the presence or absence of Scrl. *n* = 17 biological replicates. **f**, Representative 3D reconstruction (*z*-stacks) from naive T cells cultured with or without Scrl and activated with anti-CD3. Scale bars, 2 µm. **g**, Average volume of PLCγ1 clusters. *n* = 3 average volumes of at least 3 cells per image in separate fields. **h**, Representative flow cytometry plot for calcium flux using INDO1 in T cells activated with anti-CD3 and streptavidin. **i**, The percentage of T cells undergoing calcium flux. **j**, Representative flow cytometry plot for intracellular calcium flux with INDO1 in the presence of 1 mM EDTA. **k**, The percentage of T cells undergoing intracellular calcium flux. *n* = 3 technical replicates per condition. **l**,**m**, T cells were activated with anti-CD3/CD28 in the presence of DMSO or ionomycin (Iono) (125 ng ml^−1^) with or without 0.5 mM Scrl. **l**, The percentage of proliferating T cells. *n* = 3 technical replicates/condition. **m**, Intracellular cytokine staining for IFNγ and TBET. *n* = 5 technical replicates per condition. Data are mean ± s.e.m. (**d**) or mean ± s.d. (**c**,**g**,**i**,**k**,**l**,**m**). Significance was tested using unpaired (**g**,**i**,**k**,**l**) or paired (**e**) two-tailed Student’s *t*-test; one-way ANOVA with Tukey’s multiple comparison test (**m**). Data are representative of two (**f**,**g**) or three (**b**,**h**–**m**) independent experiments. Source data

**Extended Data Fig. 6:**
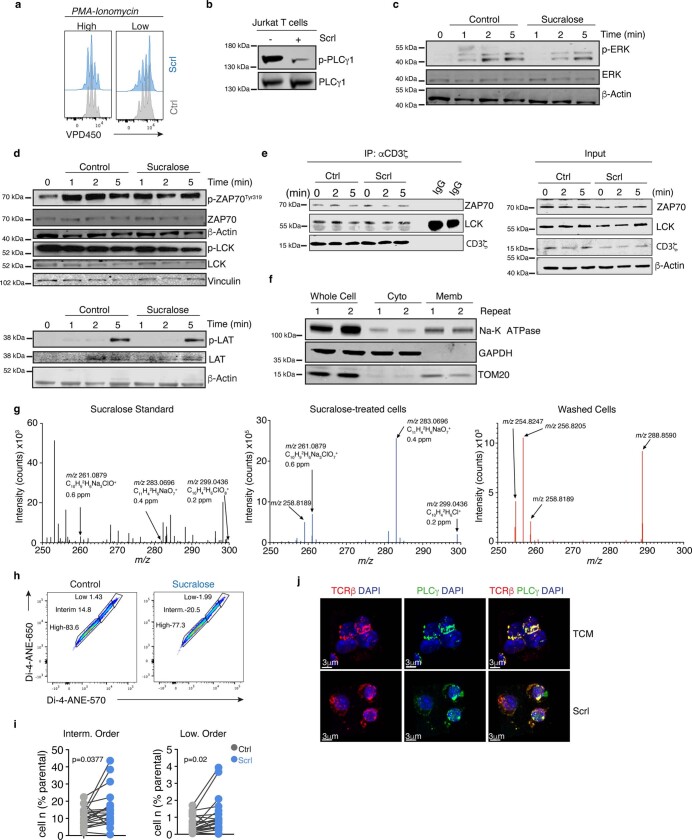
Sucralose reduces membrane order. **a**) Flow cytometry histogram overlay of T cells loaded with VDP450 and activated with either high concentration of PMA (10 μg ml^−1^) and ionomycin (500 ng ml^−1^) (left) or low concentration (1 ng ml^−1^ PMA and 50 ng ml^−1^ ionomycin) (right), respectively. **b**) Western blot analysis of Jurkat T cells cultured with or without 0.5 mM Scrl and probed for p-PLCγ1 and PLCγ1 total. (**c–d**) Western blot analysis of T cells pre-treated for 2 h with or without 0.5 mM Scrl and activated with αCD3 (5 μg ml^−1^) for 1, 2, and 5 min. Membranes were probed for (**c**) p-ERK1/2 and total ERK1/2 expression, using β-Actin as loading control. All proteins detected on the same membrane. (**d**) Identical protein lysates were probed for p-ZAP70^Tyr319^, ZAP70, and β-Actin as loading control on one membrane and p-LCK, LCK and vinculin as loading control were probed on a second membrane. Second set of protein lysates were probed for p-LAT, LAT and β-Actin as loading control on the same membrane. **e**) Immunoprecipitation (IP) of CD3ζ chain from Jurkat T cells cultured media in presence or absence of 0.5 mM Scrl for 2 days. (Left) Cells were stimulated with 5 µg ml^−1^ αCD3 for 0’, 2’, 5’ min as indicated. (Right) Western blot analysis of protein lysates used for IP showing ZAP70, LCK and CD3ζ expression with β-Actin as loading control. All proteins detected on the same membrane. **f**) Western blot analysis of protein lysates from whole cell fractions, cytoplasmic fractions and membrane fractions were probed for Na-K ATPase (membrane marker), GAPDH (cytoplasmic marker) and TOM20 (mitochondrial marker). All proteins were detected on the same membrane. **g**) Cryogenic Argon GCIB Orbitrap mass spectra acquired through the entire depth of cells until substrate signal was detected, showing the presence of fragments of deuterium labelled sucralose (d-Scrl) in a sample containing Jurkat T cells treated with 0.5 mM d-Scrl for 48 h, and subsequent absence of any sucralose-related ions in a sample containing d-Scrl treated cells which were subsequently washed with PBS to remove any traces of d-Scrl in the media. Masses annotated with chemical formulas derive from d-Scrl. Identifications were performed in comparison with a standard of pure d-Scrl. **h**) Representative flow cytometry plot of membrane orders as detected with the Di-4-ANEPPDHQ dye. Low, intermediate, and high membrane order populations are denoted. **i**) Paired analysis of Intermediate and Low membrane order of CD8^+^ T cells activated with αCD3/CD28 for 3 days with or without 0.5 mM Scrl. n = 20 biologically replicates. **j**) Representative 3D reconstruction (Z-stacks) from naïve T cells pre-treated with or without 0.5 mM Scrl, followed by TCR-crosslinking. Images show colocalization of TCRβ (red), PLCγ1 (green), and nuclei are stained with DAPI. Bar = 3 μm. Statistical significance was tested using, paired (**i**) 2-tailed student’s t-test. Data represent one of 3 independent experiments (**a–f, h–j**). Source data

To understand at which point sucralose affects TCR signalling, we activated downstream pathways of the TCR with phorbol 12-myristate 13-acetate (PMA) (to induce PKC-driven RAS activation) and ionomycin (to increase intracellular calcium). We did not detect any effect of sucralose in response to stimulation with PMA or ionomycin (Extended Data Fig. 6a); we therefore focused on earlier TCR-induced signalling events (Fig. 2a). A key response downstream of TCR stimulation is the phosphorylation and activation of PLCγ1, which cleaves phosphatidylinositol-4,5-bisphosphate (PtdInsP_2_) into inositol-1,4,5-trisphosphate (InsP_3_) and diacylglycerol^28,29^ (DAG) (Fig. 2a). Sucralose-exposed T cells showed a clear delay in PLCγ1 phosphorylation at early timepoints following TCR activation (Fig. 2b). Similarly, Jurkat T cells cultured in sucralose showed diminished PLCγ1 phosphorylation (Extended Data Fig. 6b). In primary mouse T cells, we detected slightly delayed ERK phosphorylation (Extended Data Fig. 6c), which recovered within 5 min. We did not observe differences in early events of TCR signalling, including ZAP70 and LAT activation, and in immunoprecipitation experiments, we did not observe major defects in the association of ZAP70 with CD3ζ in Jurkat cells following stimulation (Extended Data Fig. 6d,e). The principal effect of sucralose is therefore to limit PLCγ1 activation without substantially affecting other early events downstream of the TCR. We next considered mechanisms through which sucralose could impede TCR signalling to PLCγ1. We analysed whole-cell, cytosolic and membrane fractions of Jurkat T cells that had been exposed to sucralose (Extended Data Fig. 6f), finding that sucralose was associated predominantly with cell membranes (Fig. 2c). Using the mass spectrometry imaging platform cryo-OrbiSIMS, we examined the spatial distribution of sucralose in activated T cells. In line with previous work^30^, depth-profiling data indicated that sucralose did not accumulate inside the cells (Fig. 2d). Furthermore, spectroscopic data indicated that sucralose was efficiently washed off the cell surface of Jurkat T cells, suggesting that it does not interact stably with the cell membrane (Extended Data Fig. 6g). However, previous studies indicated that sucralose affects lipid packing and membrane fluidity in lipid membranes^31^. We found that T cell membranes from sucralose-treated cells were consistently shifted to a lower order that is associated with reduced responses^32^ (Fig. 2e and Extended Data Fig. 6h,i). These changes in membrane order correlated with a reduction in PLCγ1 clustering and colocalization with TCRβ on the cell surface in response to TCR stimulation (Fig 2f,g and Extended Data Fig. 6j). PLCγ1 clustering has been shown to be required for signal transduction^33^, and this defect in sucralose-treated cells could explain their incomplete activation of PLCγ1 (Fig 2b).

## Reduced intracellular TCR calcium release

**Extended Data Fig. 7:**
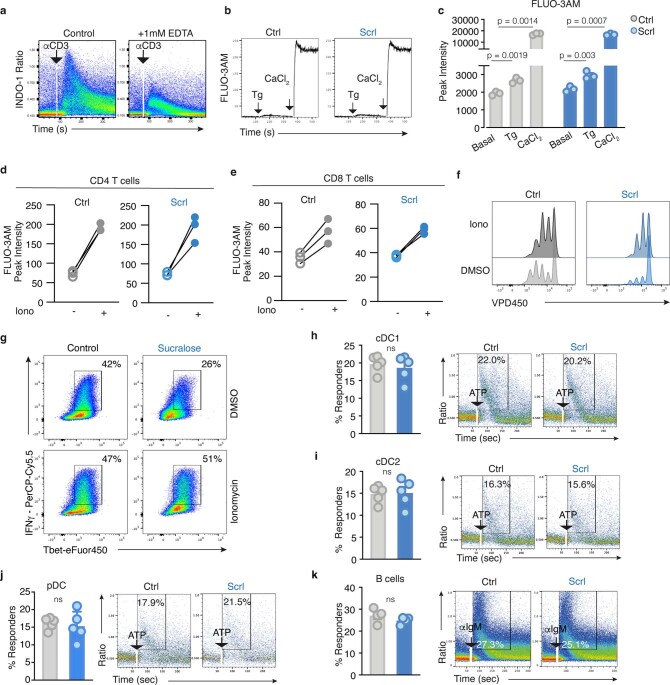
Sucralose selectively reduces TCR-induced intracellular calcium flux. **a**) Representative intracellular calcium flux in naïve CD4^+^ T cells activated with αCD3-biotin crosslinked by streptavidin with or without 1 mM EDTA. Intracellular calcium flux is plotted as INDO1 ratio over time. **b**) Representative kinetic plot measuring calcium flux with FLUO-3AM using thapsigargin 1 μM (Tg) (first arrow) followed by 2 mM calcium chloride (CaCl_2_) (second arrow) in the presence or absence of 0.5 mM Scrl. The line represents mean values with gaussian smoothing. **c**) FLUO-3AM peak intensities upon Tg and CaCl_2_ treatments in CD4^+^ T cells in media lacking calcium salt. N = 3 biological replicates per condition. The statistical significance of Tg and CaCl_2_ vs Basal was tested using a paired 2-tailed Student’s t test with p values as reported in the figure. 2-way anova with Sidak’s multiple comparison test was used to test the effect of sucralose on the 3 conditions (Basal, Tg, and CaCl_2_). No significant effect was discovered. (**d–e**) Pairwise comparison of changes in FLUO-3AM peak intensity with ionomycin treatment in CD4^+^ (**d**) and CD8^+^ (**e**). N = 3 biological replicates per condition. T cells were tested in calcium-free media with or without 0.5 mM Scrl (blue). Each dot represents a biological replicate. **f**) Representative histogram plot of VPD450 dilution of total T cells activated with αCD3 (2 μg ml^−1^) and αCD28 (1 μg ml^−1^) in the presence of DMSO or 125 ng ml^−1^ of ionomycin with or without 0.5 mM Scrl. **g**) Representative flow cytometry plot of T cells activated with αCD3 (2 μg ml^−1^) and αCD28 (1 μg ml^−1^) in the presence of DMSO or 125 ng ml^−1^ of ionomycin with or without 0.5 mM Scrl and restimulated for ICS. Cytokine production is identified as Tbet^+^IFNγ^+^ populations. (**h–j**) FLT3-ligand generated conventional DC1 (n = 3/condition, technical replicates) (**h**), cDC2 (n = 3/condition, technical replicates) (**i**) and plasmacytoid DCs (n = 3/condition, technical replicates) ( **j**) differentiated in the presence or absence of 0.5 mM Scrl. Graphs on the left show the percentage of cells undergoing calcium flux in response to 1mM ATP. The representative flow cytometry plots (right) show the INDO1 ratio over time in response to ATP. **k**) Isolated naïve B cells stained with INDO1 and activated with αIgM (20 μg ml^−1^) in presence or absence of 0.5 mM Scrl. Graph (left) represents the percentage of calcium responding cells downstream of αIgM stimulation and on the right is a flow cytometry representative plot. N = 5 technical replicates per condition. Data present as mean value ±s.d. (**c**) or ±s.e.m. (**h–k**). Data representative of 3 independent experiments (**h–k**). Statistical significance was tested using paired (**c–e**) or unpaired 2-tailed Student’s t test (**h–k**). Source data

As calcium release from intracellular stores is downstream of PLCγ1 activation^34^, we next examined whether sucralose affected calcium flux upon TCR engagement. Using flow cytometry, we found that sucralose reduced TCR-dependent calcium flux in T cells (Fig. 2h,i). Calcium is first released from internal stores into the cytosol, which is followed by uptake of extracellular calcium^35,36^. To determine which calcium source is affected by sucralose, we treated the cells with EDTA to inhibit entry of extracellular calcium (Extended Data Fig. 7a). Sucralose-treated naive T cells retained reduced TCR-dependent calcium flux compared with control cells under these conditions (Fig. 2j, k). These results therefore point to a defect in the release of intracellular calcium stores downstream of the TCR.

To test whether sucralose affected the ability of cells to store calcium, we used thapsigargin to block calcium entry into the endoplasmic reticulum. T cells treated with thapsigargin exhibited similar cytosolic calcium accumulation in the presence or absence of sucralose and, following the addition of extracellular calcium, elicited similar calcium mobilization under both conditions (Extended Data Fig. 7b,c). These results suggested that intracellular calcium stores were unaffected by sucralose; to further verify this observation, we used ionomycin in the absence of exogenous calcium to induce the release of calcium from intracellular stores. Again, we found that under these conditions, sucralose did not affect intracellular calcium release (Extended Data Fig. 7d,e). Our data indicate that sucralose affects TCR- and PLCγ1-dependent intracellular calcium release without changing overall intracellular calcium storage. In line with these observations, we were able to partially rescue proliferation and cytokine production with ionomycin (Fig. 2l,m and Extended Data Fig. 7f,g).

Calcium is an important second messenger in other immune cell types, such as dendritic cells and B cells, so we assessed whether sucralose influenced these populations. Sucralose did not impair calcium flux in in vitro-generated conventional type 1 or type 2 dendritic cells (cDC1s, cDC2s) and plasmacytoid dendritic cells (pDCs) in response to ATP^37^ (Extended Data Fig. 7h–j). Similarly, we did not detect sucralose-dependent changes in calcium responses downstream of B cell receptor engagement (Extended Data Fig. 7k). Together, these data are consistent with an ability of sucralose to selectively impair TCR-mediated intracellular calcium release and proliferation.

## In vivo tumour-specific T cell responses

**Fig. 3:**
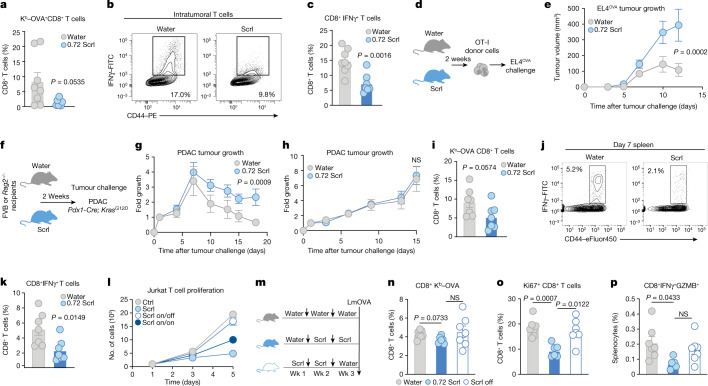
Sucralose treatment limits T cell-specific responses in vivo. **a**–**c**, CD8 antigen-specific responses to subcutaneous EL4-OVA tumour growth in mice given water (*n* = 8) or 0.72 mg ml^−1^ Scrl (*n* = 7). **a**, Quantification of intratumoral CD8–MHC tetramer (K^b^)–OVA-specific T cells. **b**, Representative flow cytometry plot of intratumoral cells re-stimulated with OVA peptide and analysed for IFNγ and CD44. **c**, Percentage OVA-specific T cells expressing IFNγ. **d**,**e**, The OT-I tumour-rejection model. Mice given water or 0.72 mg ml^−1^ Scrl (*n* = 10 per condition). **d**, Schematic overview of the model. **e**, Volumes of EL4-OVA tumours. **f**,**g**,**h**, MHC-mismatched tumour model using *Kras*^*G12D*^ pancreatic ductal adenocarcinoma (PDAC) cells in recipient mice given water (*n* = 9) or 0.72 mg ml^−1^ Scrl (*n* = 8). **f**, Schematic overview of the model. **g**, Tumour growth in FVB recipient mice. **h**, Tumour growth in *Rag2*^−/−^ recipient mice. **i**–**k**, C57BL/6J mice given water (*n* = 7) or 0.72 mg ml^−1^ Scrl (*n* = 7) challenged with LmOVA. **i**, The percentage of splenic OVA-specific CD8^+^ T cells. **j**, Representative flow cytometry plot of splenocytes re-stimulated with OVA peptide and analysed for expression of CD44 and IFNγ. **k**, The percentage of splenic CD8^+^ T cells expressing IFNγ. **l**, Representative proliferation of Jurkat T cells in T cell media (Ctrl) or exposed to acute (Scrl), chronic (Scrl on/on) or transient (Scrl on/off) 0.5 mM Scrl. *n* = 3 per condition. representative of 3 independent experiments. **m**–**p**, T cell responses at day 7 after LmOVA infection. Mice given water (*n* = 7), 0.72 mg ml^−1^ Scrl (*n* = 6) or 0.72 mg ml^−1^ Scrl for 2 weeks followed by water for one week (*n* = 7, Scrl off). **m**, Schematic experimental overview. **n**, Percentage of splenic K^b^–OVA-specific CD8^+^ T cells. **o**, The frequency of splenic Ki67^+^CD8^+^ T cells. **p**, The frequency of total IFNγ and granzyme B (GZMB) expression in splenic CD8^+^ T cells after re-stimulation with OVA peptide. Data are mean ± s.e.m. (**a**,**c**,**e**,**g**–**i**,**k**,**l**,**n**–**p**). Each dot represents a biological (**a**,**c**,**e**,**g**–**i**,**k**,**n**–**p**) or technical (**l**) replicate; data are representative of two (**e**,**g**,**i**,**k**) or three (**l**) independent experiments. Significance was tested using unpaired two-tailed Student’s *t*-test (**a**,**c**,**i**,**k**); Brown–Forsythe and Welch ANOVA test with Dunnett’s T3 comparison (**n**–**p**); two-way ANOVA **(e**,**g**,**h**). NS, not significant. Source data

**Extended Data Fig. 8:**
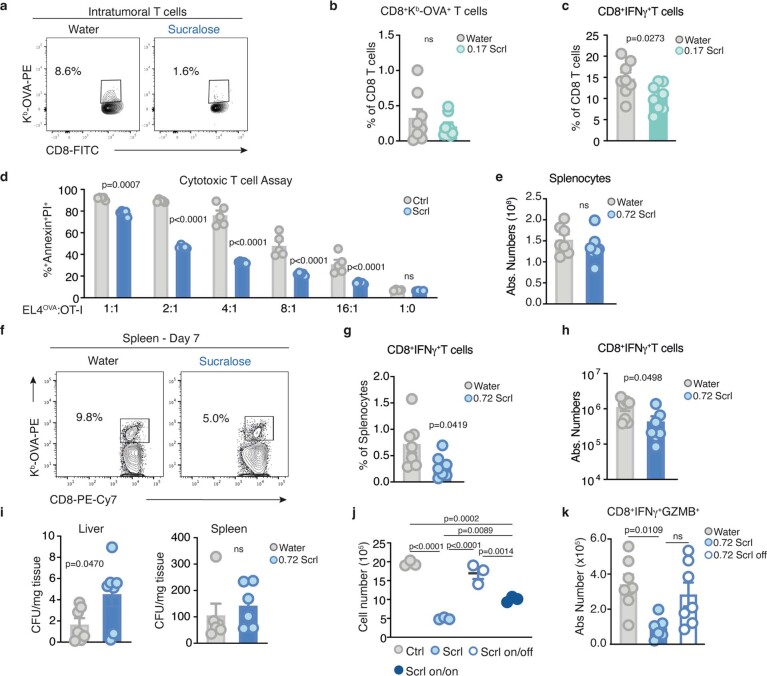
Sucralose treatment in mice reduces antigen-specifc CD8^+^ T cell responses. **a**) Representative density plot of intratumoral CD8^+^ T cells recognizing the MHC-peptide tetramer complex (K^b^-OVA) from EL4^OVA^ tumours from mice fed water or 0.72 μg ml^−1^ Scrl 10 days post challenge. (**b, c**) Mice fed water or 0.17 μg ml^−1^ Scrl . N = 8 biological replicates per group and each dot represents an individual mouse **b**) Intratumoral frequency of CD8^+^ T cells recognizing the MHC-peptide tetramer complex (K^b^-OVA) from EL4^OVA^ tumours. **c)** Intratumoral frequency of CD8^+^IFNγ^+^ T cells from tumour bearing mice post re-stimulation with the OVA peptide. **d**) Cytotoxic T cell assay of OT-I T cells activated with OVA-peptide either in TCM with (Scrl) or without (Ctrl) 0.5 mM Scrl followed by co-culture with EL4^OVA^ cancer cells. N = 5 for technical replicates. (**e–h**) Splenocytes from C57BL/6J mice fed water or 0.72 mg ml^−1^ Scrl followed by bacterial challenge with LmOVA (10^5^ CFU permouse). N = 7 biological replicates per group. **e**) Total splenocyte numbers 7 days post infection. **f**) Representative density plot of OVA-specific CD8^+^ T cells identified by K^b^-OVA and CD8^+^ surface staining. **g**) Frequency of CD8^+^ T cells re-stimulated with OVA-peptide and analyzed for IFNγ expression. **h**) Absolute numbers of splenic CD8^+^ T cells in Extended Data Fig. 8g. **i**) The bacterial load (colony forming units per mg of tissue) of the liver (left) and spleen (right) at day 3 post LmOVA (10^5^ CFU per mouse) infection of mice fed water or 0.72 mg ml^−1^ Scrl; n = 6 (spleen) or 7 (liver) of biological replicates per group. **j**) Absolute cell number of Jurkat T cells grown in media with or without Scrl as indicated at day 5. N = 3 technical replicates. **k**) Absolute splenic numbers of CD8^+^ T cells producing IFNγ and Granzyme B (GZMB) in response to OVA-peptide re-stimulation of mice infected with LmOVA (10^5^ CFU per mouse) 7 days post infection. Mice were fed water (n = 7), 0.72 mg ml^−1^ Scrl for 2 weeks (n = 6) or 0.72 mg ml^−1^ Scrl treatment for 2 weeks followed by one-week water washout (n = 7, Scrl off). Data presented as mean value ± s.e.m. (**b-e, g-k**). Each dot represents a sample derived from an individual mouse (**b**, **c**, **e**, **g**, **h**, **i, k**). Statistical significance was tested using single or multiple unpaired 2-tailed Student’s t test (**b-e, g-i**) or a One-way ANOVA with a Tukey’s Multiple Comparison ( **j, k**). Data representative of 2 (**d, e, i, k**) or 3 ( **j**) independent experiments. Source data

To expand our in vitro observations, we examined the effects of sucralose on tumour-specific T cell responses in vivo. Using the model antigen ovalbumin (OVA) to induce a major histocompatibility complex type I (MHCI)-restricted CD8^+^ T cell response^38^, we measured tumour-specific responses against subcutaneous EL4 cancer cells expressing OVA (EL4-OVA cells). There were variable degrees of OVA-specific T cell infiltration, but this was consistently lower in the tumours derived from mice given 0.72 mg ml^−1^ sucralose (Fig. 3a and Extended Data Fig. 8a). Re-stimulation of the tumour infiltrates with an OVA peptide (SIINFEKL) showed dampened IFNγ production in CD8^+^ T_eff_ cells from sucralose-exposed mice (Fig. 3b,c). Although mice treated with 0.17 mg ml^−1^ sucralose displayed no change in antigen-specific T cells, we observed a significant, but less pronounced, reduction in the function of these cells (Extended Data Fig. 8b,c). To further test antigen-specific CD8^+^ T cell responses, we adoptively transferred CD8^+^ OT-I donor T cells that recognize OVA into recipient mice given 0.72 mg ml^−1^ sucralose or water followed by EL4-OVA challenge (Fig. 3d). Consistent with weakened T cell function, we observed increased tumour growth and reduced rejection in mice treated with sucralose (Fig. 3e). Furthermore, OT-I cells activated in vitro in the presence of sucralose displayed decreased cytotoxic activity against EL4-OVA cells (Extended Data Fig. 8d). In a third model, we extended previous work showing that the rejection of infrared fluorescent protein (iRFP)-expressing *Pdx1-Kras*^*G12D*^ pancreatic tumour cells injected into MHC-mismatched recipients is dependent on a T cell response^39^ (Fig. 3f). We observed a significant delay in tumour rejection in mice treated with 0.72 mg ml^−1^ sucralose compared with control mice (Fig. 3g). This effect of sucralose was T cell-specific, as pancreatic tumour cells grew at equivalent rates in T cell-deficient mice given water or sucralose (Fig. 3h).

## Effect of sucralose on response to infection

We also assessed the effect of sucralose on CD8^+^ T_eff_ responses in an infection model by challenging wild-type mice with Gram-positive *Listeria monocytogenes* expressing OVA (LmOVA). Treatment with 0.72 mg ml^−1^ sucralose did not affect splenocyte numbers at day 7 after infection (Extended Data Fig. 8e), but caused a reduction in the frequency of splenic OVA-specific CD8^+^ T cells (Fig. 3i and Extended Data Fig. 8f). Furthermore, SIINFEKL re-stimulation of infected splenocytes from day 7 revealed a significant decrease in the frequency and number of CD8^+^ T cells producing IFNγ (Fig. 3j,k and Extended Data Fig. 8g,h) from sucralose-treated mice, suggesting impaired function. In line with reduced cytokine production, we observed increased bacterial load in the liver at day 3 after infection, although this was not evident in the spleen (Extended Data Fig. 8i). To investigate the permanence of the sucralose effect, we measured the proliferation of Jurkat T cells pre-cultured in sucralose for 2 weeks. In this model, removal of sucralose resulted in the recovery of the normal proliferation rate, suggesting that the response to sucralose is reversable (Fig. 3l and Extended Data Fig. 8j). To confirm this in vivo, we used the LmOVA model to show that removal of sucralose one week before the challenge (Fig. 3m) partially rescued the development of antigen-specific T cells, their proliferation and cytotoxic function (Fig. 3n–p and Extended Data Fig. 8k).

## Sucralose mitigates autoimmune T cell responses

**Fig. 4:**
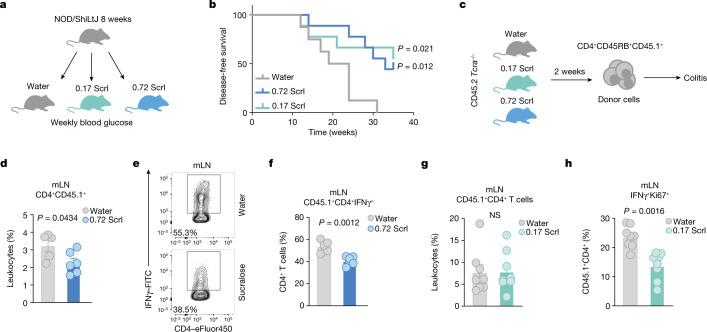
Sucralose treatment dampens T cell-mediated inflammation in models of autoimmunity. **a**,**b**, NOD/ShiLtJ type 1 diabetes model in mice given water (*n* = 8), 0.72 mg ml^−1^ Scrl (*n* = 9) or 0.17 mg ml^−1^ Scrl (*n* = 9). **a**, Schematic of the model. **b**, Disease-free survival. **c**, Schematic of the T cell-induced colitis model. **d**–**f**, CD45.2 *Tcra*^−/−^ recipient mice treated with (*n* = 6) or without (*n* = 5) 0.72 mg ml^−1^ Scrl. **d**, The percentage of congenic CD45.1^+^CD4^+^ donor T cells in the mLN 3 weeks after transplantation. **e**, Representative flow cytometry plot of lymphocytes from the mLN that were re-stimulated and analysed for the expression of IFNγ and CD4. **f**, The percentage of CD4^+^CD45.1^+^ donor cells in the mLN that express IFNγ. **g**,**h**, Analysis of mLNs from recipient CD45.2 *Tcra*^−/−^ mice given water (*n* = 8) or 0.17 mg ml^−1^ Scrl (*n* = 8). **g**, The total frequency of CD45.1^+^CD4^+^ donor T cells. **h**, The frequency of CD4^+^ donor T cells expressing Ki67 and IFNγ. Data are mean ± s.e.m. (**d**,**f**–**h**). Each dot (**d**,**f**–**h**) represents a biological replicate; data are representative of two (**d**–**f**) or three (**g**,**h**) independent experiments. Significance was tested using unpaired two-tailed Student’s *t*-test (**d**,**f**–**h**) and log rank Mantel–Cox test (**b**) for either dose of Scrl versus water. Source data

**Extended Data Fig. 9:**
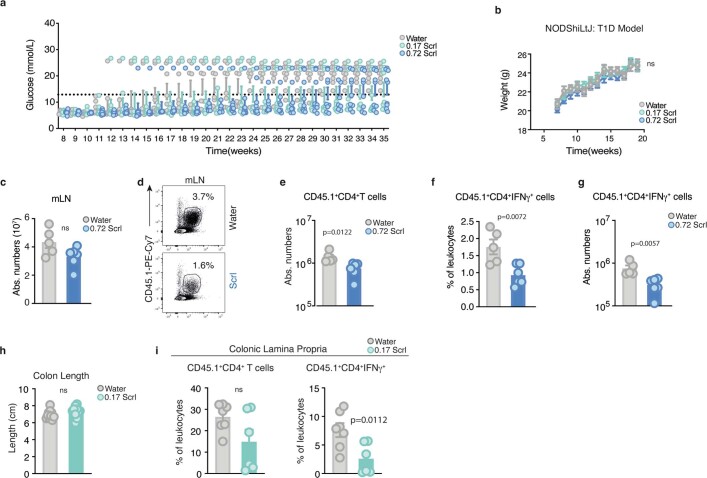
Sucralose delays the onset of T1D and reduces inflammatory T cells in a T-cell induced colitis model in mice. **a-b**). NOD/ShiLtJ mice fed with either water (n = 8), 0.17 or 0.72 mg ml^−1^ Scrl (n = 9 for each dose) . **a**) Weekly blood glucose measurements. **b**) Weekly weight measurements. (**c–g**) T cell-induced colitis model of CD4^+^CD45RB^+^CD45.1^+^ congenic T cells transferred into CD45.2 TCRα^−/−^ recipients that were fed either water (n = 5) or 0.72 mg ml^−1^ Scrl (n = 5). Inflammation was assessed at 21 days post T cell transfer. **c**) Total numbers of mesenteric lymph node (mLN) leukocytes. **d**) Representative density plot of the percentage of CD4^+^ CD45.1^+^ donor cells in the mLN of TCRα^−/−^ recipients. **e**) Total numbers of donor congenic donor T cells in the mLN. **f**) Percentage of congenic CD4^+^ T cells producing IFNγ^+^ in the mLN. **g**) The absolute number of congenic CD4^+^IFNγ^+^ T cells within the mLN. (**h–i**) T-cell induced colitis model of CD45.1 congenic naive T cells adoptively transferred into CD45.2 TCRα^−/−^ recipients that were fed either water or 0.17 mg ml^−1^ sucralose. **h**) Colon length at 21 days post transfer (n = 8/group). **i**) Frequency of CD45.1 CD4+ donor T cells producing IFNγ in the colonic lamina propria at 21 days post transfer. Water (n = 7) versus 0.17  mg ml^−1^ sucralose (n = 6). Data presented as mean value ±s.e.m. (**a–c**, **e–i**). Each dot represents an individual mouse (**a**, **c, e–i**). Statistical significance was tested using unpaired 2-tailed Student’s t test (**c**, **e–i**) and mixed-effects model (REML) (**b**). Data are representative of 2 (**c, e–g**) or 3 (**i, h**) individual experiments. Source data

Our observations that sucralose can dampen T cell responses in vitro and in vivo prompted us to determine whether sucralose could also have therapeutic value by limiting T cell-mediated autoimmunity. Female NOD/ShiLtJ mice provide a spontaneous model of type 1 diabetes characterized by hyperglycaemia and insulitis between 12 and 30 weeks of age, which is caused by T cell-mediated destruction of the pancreatic islets^40,41^. Mice given either sucralose dose showed lower frequencies of hyperglycaemia and delayed development of type 1 diabetes, an effect that was independent of weight gain (Fig. 4a,b and Extended Data Fig. 9a,b). As a second model of T cell-mediated autoimmunity, we measured T cell-induced colitis by adoptively transferring immunodeficient CD45.2 *Tcra*^*−/−*^ mice with congenic CD45.1 naive CD4^+^ T cells^42^ (Fig. 4c). In mice treated with 0.72 mg ml^−1^ sucralose, we observed reduced frequencies and numbers of donor CD45.1^+^CD4^+^ T cells, with no effect on total mesenteric lymph node (mLN) leukocytes (Fig. 4d and Extended Data Fig. 9c–e). Re-stimulation of the mLN at day 21 showed lower frequencies and reduced total numbers of pro-inflammatory IFNγ-producing CD4^+^ T cells in sucralose-treated mice (Fig. 4e,f and Extended Data Fig. 9f,g). Lowering the dose of sucralose led to a reduction in proliferating IFNγ-producing CD4^+^ T cells, without affecting the frequencies of donor CD4^+^ T cells and colon length at day 21 (Fig.4g,h and Extended Data Fig. 9h,i). These data suggest that supplementation with sucralose mitigates T cell-mediated autoimmune responses.

In sum, this work has revealed an unexpected role for high doses of sucralose in modulating immunity by affecting T cell proliferation and effector function. Notably, although the doses of sucralose used in this study are clearly higher than those resulting from normal human dietary consumption of sucralose-sweetened drinks and foods, they are relevant to the ADI recommendation when BSA-adjusted for mice. Our findings do not, therefore, provide evidence that normal sucralose intake is immunosuppressive, but they do demonstrate that at high (but achievable) doses, sucralose has an unexpected effect on T cell responses and functions in autoimmune, infection and tumour models. Our observation that sucralose lowers membrane order and reduces calcium flux is consistent with previous studies^43^, suggesting that the sucralose effect on the plasma membrane could drive the defect in PLCγ1 activation and calcium release. However, the precise mechanistic details of how sucralose affects TCR signalling remain to be determined. Further experiments using primary T cells and super high-resolution microscopy are necessary to better evaluate the nanocluster formation upon TCR engagement, assess the recruitment of LAT in the presence of sucralose and establish a causal link between these observations. Although our results support a direct effect of sucralose on TCR signalling, we cannot exclude the possibility that sucralose may also affect T cells through additional mechanisms, such as epigenetic changes in response to long-term sucralose exposure or an ability to modulate taste receptors that are not shared with other sweeteners. Furthermore, although we did not observe major changes in the microbiome, such alterations have been noted previously^11^ and are likely to contribute to the overall response to sucralose intake. Surprisingly, our data suggest that sucralose does not impede calcium signalling in other immune cell types such as B cells or dendritic cells. It is possible that the membrane composition of T cells makes them particularly sensitive to sucralose and it remains to be determined whether sucralose affects other cell types, including other immune cells, in conditions not tested in this study. In conclusion, our study adds to the evidence that sucralose is not an inert molecule and may affect human health. If translatable, our work suggests that treatment with doses of sucralose similar to those used in this study may be beneficial for various conditions arising from unrestrained T cell activity.

## Methods

Antibodies used in this study are listed in the Supplementary Information.

### Mice and in vivo models

For all mouse experiments, at least four mice per group were used. For more complex models, we used more animals to compensate for the increased expected variability. Mice were randomly assigned to a treatment group. For metabolic phenotyping mice were divided in groups after measuring starting body weight to have weight-matched cohorts.

C57BL/6J, FVB/NJ*, Tcra*^−/−^, and *Rag2*^−/−^, *Rag2*^−/−^ OT-I and B6.SJL-*Ptprc*^*a*^*Pepc*^*b*^/BoyCrl mice were bred and housed at the Francis Crick Institute animal facility. NOD/ShiLtJ mice were purchased from Charles River Laboratory. Animal experiments were subject to ethical review by the Francis Crick Animal Welfare and Ethical Review Body and regulation by the UK Home Office project licence P319AE968. All mice were housed under conditions in line with the UK Home Office guidelines. Mice were kept in a 12-h day:night cycle starting at 07:00 until 19:00. Food and water were available ad libitum and rooms were kept at 21 °C at 55% humidity. For sucralose treatment, sucralose (Merck) was dissolved in drinking water at the final concentration of 0.72 or 0.17 mg ml^−1^ as indicated. The sucralose solution was filtered through a 0.2 μM filter before being added to drinking bottles. Sucralose solutions were replaced once or twice a week and sucralose consumption was measured by the change in weight of the drinking bottles. A similar procedure was followed to prepare any solution that was given to mice. All procedures were performed following the Animals (scientific procedures) Act 1986 and the EU Directive 2010.

### Physiological measures

Food intake, solution intake and body weight.

Individually caged male C57BL/6J 7–8-week-old littermates were used for measurements of body weight, food intake and solution intake. Body weight and food intake were measured weekly. Solution intake was measured twice weekly by measuring the weight of the solutions. Fresh solutions were provided after every measurement every 3–4 days.

### Metabolic phenotyping

Male mice were individually housed in metabolic cages of a combined indirect calorimetry system (TSE PhenoMaster, TSE systems), and after a 48 h acclimatization period, we continually measured O_2_ consumption, CO_2_ production, respiratory exchange ratio (RER), energy expenditure, and locomotor activity (that is, horizontal and vertical beam breaks) of individual mice in 15-min intervals for a total of 108 h. Body composition was measured using Echo-MRI device (Echo-MRI). This animal study was approved by the Animal Ethics Committee of the government of Upper Bavaria (Germany).

### Glucose tolerance test

Individually housed male C57BL/6J mice aged 7–8 weeks were given either water or the different sweeteners as indicated for more than 12 weeks. Mice were food deprived for 6 h before receiving an oral gavage of 2 mg kg^−1^ of glucose (Merck, 158968). Blood glucose was measured using a glucometer (Accu-CHEK) before the oral gavage and every 30 min up to 120 min post gavage.

### Gut microbiome analysis

Faecal samples were freshly collected from individually caged littermates C57BL/6J male mice aged 8 weeks exposed to the different drinking solutions as indicated. Samples were collected after 2 and 12 weeks of treatment. At least two faecal pellets were collected for each mouse, snap frozen in liquid nitrogen and stored at −80 °C until extraction. Faecal DNA was isolated using QIAamp PowerFecal DNA KIT (Qiagen, 12830-50). The 16S amplicons were prepared for sequencing by indexing PCR by a reduced-volume reaction based on the Illumina 16S Metagenomics Sequencing Protocol (15044223 Rev. B). In brief, 2 μl of each sample was combined with 3.25 μl H_2_O, 6.25 μl 2× NEB Q5 High-Fidelity DNA Polymerase Master Mix (M0492L) and 1 μl of a unique 10 µM UDI primer from the set Nextera IDT-8nt (384 Indexes). Samples were incubated at 95 °C for 3 min, followed by 10 cycles of 95 °C for 30 s, 55 °C for 30 s, 72 °C for 30 s, followed by a final extension at 72 °C for 5 min.

A bead-based clean-up was carried out with Beckman Coulter SPRIselect (B23319) in a 0.8× ratio. The quality of the purified libraries was assessed using a D1000 ScreenTape on an Agilent 24200 TapeStation. Libraries were sequenced on the Illumina MiSeq platform. 2× 300 paired-end reads were produced using the 600 cycle MiSeq Reagent kit v3. Libraries were loaded at 8 pM concentration with 20% PhiX.

The fastq files were processed using DADA2 (v1.18)^44^, truncating the forward (respectively reverse) reads to 280 and 210 bases and trimming them by 17 and 21 bases, respectively, with a maximum of two expected errors. Taxa and species assignment was carried out using v132 of the SILVA database. The processed data were then analysed in R v4.0.3 (ref. ^45^) with the phyloseq package^46^ aggregating the count data to the genus level. DESeq2 (v1.30 (ref. ^47^)) was used to estimate the log fold changes and *P* values between experimental groups whilst accounting for an observed batch effect that crossed the experimental groups.

### Homeostatic proliferation

C57BL/6J *Rag2*^−/−^ male and female mice aged 8–10 weeks were provided either water or sucralose (0.72 mg ml^−1^ or 0.17 mg ml^−1^) ad libitum for 2 weeks. CFSE-stained lymphocytes from C57BL/6J donors (stained according to manufacturer’s instructions, BioLegend) were then injected intravenously at 1 × 10^6^ cells per mouse in PBS. At day 3 post injection spleens were assessed for CFSE dilution of donor T cells by flow cytometry.

### Tumour challenge and rejection models

Mice were exposed to either water or sucralose (0.72 mg ml^−1^) in the drinking water ad libitum 2 weeks before tumour challenge and exposed to solutions until the end of the experiment.

*Pdx1-cre*; *Kras*^*G12D*^ PDAC cells expressing iRFP^39^ were subcutaneously injected into the left flank of FVB/NJ or C57BL/6J *Rag2*^−/−^ mice at 1 × 10^6^ cells per mouse in PBS. Growth was monitored by in vivo imaging and mice were taken at a humane endpoint as dictated by the UK Home Office and the animal license. Humane endpoints were maximum tumour size of 1.2 cm, tumour ulceration or 10% weight loss, as authorized in the UK Home Office project licence P319AE968. None of these limits were exceeded.

For in vivo imaging, iRFP fluorescence was measured (excitation: 685 nm and emission: 730 nm) using the LiCOR Odyssey Pearl Imager and analysed with Image Studio v5 (LiCOR).

EL4-OVA cells (1 × 10^6^ cells per mouse) resuspended in PBS were subcutaneously injected into the left flank of C57BL/6J recipient mice either exposed to water or sucralose (0.72 mg ml^−1^ or 0.17 mg ml^−1^) 2 weeks before challenge and until the end of the experiment, 10 days post injection. Tumours were digested and analysed for OVA-specific CD8^+^ T cells and IFNγ production in response to SIINFEKL peptide stimulation, followed by intracellular cytokine staining. Samples were acquired on the BD LSR Fortessa and on the BD FACSymphony. Flow cytometry data were analysed using FlowJo v10 (TreeStar).

The OT-I tumour-rejection assay was performed by exposing C57BL/6J recipients to either water or sucralose (0.72 mg ml^−1^) for 2 weeks, followed by intravenous injection of 0.3 × 10^6^ TCR-transgenic OT-I T cells from *Rag2*^−/−^ OT-I donor mice. The next day mice were injected subcutaneously in the left flank with EL4-OVA cancers cells (1 × 10^6^ cells per mouse). Growth was monitored by calliper measurements and mice were taken at a humane endpoint as dictated by the UK Home Office and the animal license. Tumour volume was calculated as volume = (length × width^2^)/2, with the length as the longest diameter and width measured as the perpendicular tumour diameter. Humane endpoints were maximum tumour size of 1.2 cm, tumours ulceration or 10% weight loss, as authorized in the UK Home Office project licence P319AE968. None of these limits were exceeded.

### *L. monocytogenes* infection

Male C57BL/6J mice (aged 8–10 weeks) were given either water or sucralose (0.72 mg ml^−1^) for 2 weeks before infection and remained on the solutions throughout the experiment. The sucralose washout experiment involved mice exposed to water for 3 weeks, mice exposed to 0.72 mg ml^−1^ of sucralose 2 weeks before injection and maintained on sucralose until the end of the experiment, and the washout group, which was provided sucralose for 2 weeks followed by water for 1 week before LmOVA challenge (Fig. 3m). In brief, mice were injected intravenously with a sublethal dose of LmOVA (1 × 10^5^ colony-forming units per mouse) and were euthanized 7 days post infection. Splenocytes were analysed for the presence of OVA-specific CD8^+^ T cells (using fluorochrome conjugated MHC tetramer complex, Baylor College of Medicine, USA) and cytokine production by CD8^+^ T cells in response SIINFEKL peptide re-stimulation, followed by intracellular cytokine staining. Samples were acquired on the BD LSR Fortessa and on the BD FACSymphony. Flow cytometry data were analysed using FlowJo v10 (TreeStar).

Bacterial load was measured at day 3 post infection. Spleen and liver were isolated and weighed, followed by tissue disruption. Spleen and liver homogenates were resuspended in PBS and 1/10 serial dilutions were performed in PBS. Fifty microlitres of each serial dilution was plated on brain heart infusion plates and placed into a bacterial incubator at 37 °C overnight. The number of colony-forming units were counted the following day and normalized to tissue weight.

### T cell-induced colitis model

Six- to eight-week-old male C57BL/6J *Tcra*^−/−^ mice were given either water or sucralose (0.72 mg ml^−1^ or 0.17 mg ml^−1^) ad libitum for 2 weeks before T cell transfer and until the end of the experiment. 0.5 × 10^6^ cells congenic CD45.1^+^CD4^+^CD45RB^+^ T cells were injected per mouse. Inflammation at day 21 was assessed by intracellular cytokine staining followed by flow cytometric analysis. Humane endpoints were maximum weight loss 15% and chronic diarrhoea as authorized in the UK Home Office project licence P319AE968. None of these limits were exceeded.

### Type 1 diabetes model

Female NOD/ShiLtJ were purchased from Charles River laboratory. Starting from 8 weeks of age, age-matched and weight-matched mice were given either water or sucralose (either 0.72 mg ml^−1^ or 0.17 mg ml^−1^). To monitor the development of type 1 diabetes, blood glucose was monitored once a week using a glucometer (Accu-CHEK). Mice with a non-fasting glucose level exceeding 13.9 mmol l^−1^ were measured a second time the following day and mice with consecutive blood glucose exceeding 13.9 mmol l^−1^ were considered diabetic. Humane endpoints were defined as consecutive non-fasting blood glucose measurements exceeding 13.9 mmol l^−1^ as authorized in the UK Home Office project licence P319AE968. None of these limits were exceeded.

### LPS-induced systemic inflammation model

Male C57BL/6J mice aged 8–10 weeks were randomly assigned either to water or sucralose (0.72 mg ml^−1^) for 2 weeks before LPS challenge and until the end of the experiment. Mice were injected intraperitoneally with LPS from *Escherichia coli* (0111:B4 Sigma L4391) at a dose of 0.1 mg kg^−1^. Mice were euthanized 3 h after challenge and blood was collected by cardiac puncture in EDTA-coated tubes.

### In vivo proliferation of peritoneal macrophages

C57BL/6J mice (one cohort of male mice aged 8–10 weeks and one cohort of female mice aged 8–10 weeks) were given sucralose (0.72 mg ml^−1^) or water ad libitum for 2 weeks and until the end of the experiment. Mice were then injected intraperitoneally with long-lasting IL-4 complex (5 µg IL-4 (Peprotech): 25 µg ml^−1^ anti-IL-4 monoclonal antibody, clone 11B11; BioXcell) or PBS (control) every second day. Mice were sacrificed after the second injection. Peritoneal macrophages were collected by injecting 5 ml of PBS into the peritoneal cavity. The exudate was collected and stained for flow cytometry. Samples were acquired on the BD LSR Fortessa. Flow cytometry data were analysed using FlowJo v10 (TreeStar).

### sRBC cell immunization

Female C57BL/6J mice (aged 8–10 weeks) were randomly assigned to 2 groups: water treatment or sucralose treatment (0.72 mg ml^−1^). Mice were exposed to sucralose for 2 weeks before immunization and until the end of the experiment. sRBCs (Antibodies Online, cat. ABIN770402) were prepared in HBSS and mice were intraperitoneally immunized with 2 × 10^9^ sRBC in PBS. Germinal centre B cells were analysed in the spleen at day 7 post immunization by cell surface staining of B220 (clone RA3-6B2), GL-7 (clone GL7), and CD95 (clone SA367H8). Samples were acquired on the BD LSR Fortessa and on the BD FACSymphony. Flow cytometry data were analysed using FlowJo v10 (TreeStar).

### Human CD8^+^ T cell isolation and culture

Human peripheral blood was collected from healthy, non-fasted individuals into heparinized Vacuettes (Greiner Bio-One). Mononuclear cells were isolated by layering whole blood (1:1) onto Lymphoprep (StemCell Technologies) and centrifuged at 805*g* for 20 min at room temperature. Human CD8^+^ T cells were isolated downstream using magnetic microbeads (Miltenyi Biotec; cat. 130-096-495). Isolated CD8^+^ T cells (1.0 × 10^6^ ml^−1^) were stained with CFSE (BioLegend) and activated with plate-bound anti-CD3 (2 μg ml^−1^; HIT3a, BioLegend) and free anti-CD28 (20 μg ml^−1^; CD28.2, BioLegend) in the presence or absence of NaS, AceK and sucralose (0.5 mM) in IMDM (Gibco) at 37 °C in 5% CO_2_-in-air for 3 days. After 3 h the media was supplemented with 10% hyclone fetal calf serum. After 72 h, cells were collected and stained with viability dye DRAQ7 (BioStatus). Cells were acquired (Novocyte, Agilent) and analysis performed using FlowJo version 10 (TreeStar).

Participants were recruited from the staff and student populations at Swansea University, Wales UK. Potential participants responded to ethics committee approved advertising by contacting the local clinical research facility. The clinical research facility oversaw recruitment through informed written consent in response to an ethically approved participant information sheet that explained the study. Participant recruitment was conducted by the Joint Clinical Research Facility at Swansea University with no selection bias. Informed written consent and ethical approval was obtained from Wales Research Ethics Committee 6 (13/WA/0190).

### Murine T cell isolation and cell culture

CD8^+^ T cells and CD4^+^ T cells were isolated from spleens and peripheral lymph nodes, prepared into single-cell suspensions, and lysed for red blood cells (10× RBC lysis buffer, Biolegend). T cells were isolated by negative isolation kits (StemCell Technologies) and following manufacturer’s protocol. T cells were then activated and cultured as previously described^48^ using plate-bound anti-CD3 and anti-CD28 antibodies in T cell medium (TCM) (IMDM, 10% fetal bovine serum (FBS), 1% penicillin-streptomycin and 50 μM β-mercaptoethanol).

Mouse *Pdx1-Kras*^*G12D*^ pancreatic cancer cell line was derived from the primary pancreatic tumours from the *Pdx1-cre* pancreatic cancer model. Cells were maintained in culture in DMEM, 10% FBS, and 1% penicillin-streptomycin. The EL4-OVA thymoma cell line was maintained in TCM with 0.4 mg ml^−1^ of G418 (Roche Diagnostic GmbH). All cells were incubated at 37 °C and 5% CO_2_ humidified incubators.

### T cell functional assays

#### T cell proliferation assay

Isolated naive T cells were stained with CFSE from BioLegend or with the VPD450 dye (BD Horizon) following the manufacturer’s protocol. T cells were then activated with anti-CD3 clone 145-2c11 (2 μg ml^−1^) and anti-CD28 clone 37.51 (1 μg ml^−1^), unless otherwise specified, with or without the indicated sweeteners for 3 days in a 37 °C, 5% CO_2_ humidified incubator followed by flow cytometry analysis. For IL-2 (Peprotech) supplementation, IL-2 at 20 ng ml^−1^ was added to selected wells in the presence of anti-CD3/CD28. Ionomycin-rescue experiments were conducted by adding an additional 125 μg ml^−1^ of ionomycin (Sigma) in the presence of anti-CD3/CD28. All functional grade antibodies were purchased from eBioscience, Thermo Fisher Scientific.

TCR-independent proliferation was achieved either by supplementing the media with 100 ng ml^−1^ of IL-2 or with a high dose combination of PMA (10 ng ml^−1^) and ionomycin (500 ng ml^−1^) or low dose of PMA (1 ng ml^−1^) and ionomycin (50 ng ml^−1^). T cells were allowed to proliferate for 3–4 days.

#### T cell differentiation assays

T_H_1 cells. Naive CD4^+^ T cells were isolated using the manufacturer’s instructions and activated by seeding 2 × 10^6^ cells per well in a 24-well tissue culture plate coated with anti-CD3 (5 μg ml^−1^) and anti-CD28 (2 μg ml^−1^). TCM was supplemented with IL-2 (20 ng ml^−1^, Peptrotech), IL-12 (40 ng ml^−1^, Peptrotech), and anti-IL-4 (BioLegend, clone 11B11, 504102). CD4^+^ T cells were re-stimulated with PMA–ionomycin–GolgiStop cocktail followed by surface and intracellular cytokine staining for IFNγ (clone XMG1.2) and TBET (clone 4B10) 3 days post differentiation.

CD8^+^ T_eff_ cells. Naive CD8^+^ T cells were isolated using the manufacturer’s protocol and activated using 250,000 cells per well of a 96-well plate coated with anti-CD3 (5 μg ml^−1^) and anti-CD28 (2 μg ml^−1^). TCM was supplemented with IL-2 (20 ng ml^−1^). CD8^+^ T cells were re-stimulated, followed by surface staining and intracellular cytokine staining for IFNγ expression.

#### Cytotoxic T cell assay

OVA-specific CD8^+^ T_eff_ cells from *Rag2*^−/−^ TCR-transgenic OT-I mice were expanded in vitro with the SIINFEKL peptide (10 μg ml^−1^) in TCM with or without 0.5 mM sucralose for 3 days. Live lymphocytes were selected using the Lympholyte M cell separation density gradient centrifugation method (CEDARLANE LABS) and then cocultured with EL4-OVA cells stained with VPD450 (BD Biosciences) starting at a 1:1 ratio and serially diluted to 32:1 (EL4-OVA:OT-I). Percentage of dead EL4-OVA cells was assessed by annexin V and propidium iodide (Invitrogen, eBioscience) staining as per the manufacturer’s instructions. Samples were acquired on the BD LSR Fortessa. Flow cytometry data was analysed using FlowJo v10 (TreeStar).

### Immunofluorescence and 3D reconstruction

13 mm borosilicate glass coverslips (thickness 1.5 mm, VWR) were placed in a 24-well plate and coated with 50 mg ml^−1^ of poly-d-lysine (Sigma) for 30 min at room temperature, followed by one water wash, and allowed to air dry. Two million naive T cells were gently placed over the coated slides and allowed to attach in a humidified 37 °C incubator for 30 min. T cells were activated with anti-CD3–biotin (5 mg ml^−1^) and streptavidin (20 mg ml^−1^) for 10 min at 37 °C. Unattached cells were aspirated and 4% PFA was added, and cells fixed for 10 min at room temperature, followed by one PBS wash and aspiration. Samples were blocked with 0.4% Triton X-100/PBS/10% bovine serum albumin solution. Primary antibody for PLCγ1 (Santa Cruz, clone E-2, sc7290) was diluted at 1:300 in the staining buffer (0.4% Triton X-100/PBS/2% bovine serum albumin) and incubated in the dark at room temperature for 1 h. After a PBS wash, fluorescent secondary antibody (Alexa488 Goat primary antibody to mouse IgG, Abcam 150113) was diluted at 1:500 in staining buffer and incubated in the dark at room temperature for a further 1 h. After a PBS wash, samples were incubated with DAPI solution (1:10,000 dilution in PBS; BD Pharmingen) for 5 min at room temperature. Slides were washed once with PBS, inverted and placed on antifade mounting media (VECTASHIELD) on superfrost microscope slides (Thermo Scientific, 12372098). Slides were sealed with CoverGrip coverslip sealant. Images were taken on a Zeiss Upright 710 using the ZEN (v2.3) program with a 63× oil objective and 1.4 NA. The 488 and 405 nm lasers were used for excitation and *z*-stacks were collected as 16 bits per pixel (average size of 240 × 240 pixels). Data were analysed using Imaris v9.5.1 software using the volume application.

### Bone marrow-derived macrophages and LPS stimulation in vitro

Bone marrow-derived macrophages were generated from femurs flushed with PBS. Red blood cells were lysed using 10× RBC lysis buffer (BioLegend), and remaining cells were plated on petri dishes at 5 × 10^6^ cells per 10 cm dish in IMDM supplemented with 10% FBS, 1% pen/strep, 50 μM β-mercaptoethanol and 25 ng ml^−1^ of M-CSF (Peprotech). Five days after differentiation, sweeteners were added for an additional 2 days at final concentration of 0.5 mM. LPS (Sigma *E. coli* O111:B4 LPS25) was added at day 7 post differentiation at a final concentration of 1 ng ml^−1^ and BD GolgiStop (1:1340 dilution) for 4 h followed by surface and intracellular cytokine staining for tumour necrosis factor (TNF) (clone MP6-XT22) and IL-1β-pro (clone NJTEN3). Samples were acquired on the BD LSR Fortessa and on the BD FACSymphony. Flow cytometry data was analysed using FlowJo v10 (TreeStar).

### Flow cytometry

Single-cell suspensions were stained for surface markers in PBS for 20 min at 4^o^C. Intracellular proteins (cytokines) were assessed using the FOXP3/Transcription staining buffer set (Invitrogen, eBioscience) following the manufacturer’s instructions. Cells were permeabilized for 30 min and stained for intracellular proteins for a minimum of 1 h at 4 °C. Fluorochromes were purchased from BioLegend, eBioscience (ThermoFischer Scientific), BD Pharmigen, or TONBO Scientific. All surface flurorescent antibodies were used at a dilution of 1:300. Re-stimulation was performed using PMA (Sigma-Aldrich), ionomycin (Sigma-Aldrich) and GolgiStop (BD Biosciences) for 4 h as previously described^49^. For antigen-specific response using ovalbumin, SIINFEKL (OVA_257–264_ produced by the Peptide Chemistry Facility, Francis Crick Institute) peptide was used for re-stimulation of single-cell suspensions (10 µg ml^−1^ with GolgiStop (BD Biosciences)) for 6 h followed by surface staining and intracellular cytokine staining. All intracellular antibodies were used at a dilution of 1:250. OVA-specific CD8^+^ T cells were distinguished using the K^b^–OVA-PE staining (Baylor College of Medicine, USA) and surface markers for 30 min, followed by immediate acquisition. Dead cells were distinguished using the fixable viability dye efluor780 from Invitrogen, eBioscience. Single-cell suspensions were fixed and permeabilized using the FOXP3 Transcription staining buffer set (Invitrogen, eBioscience). Samples were acquired on the BD LSR Fortessa and on the BD FACSymphony. Flow cytometry data were analysed using FlowJo (TreeStar).

### Calcium flux assays

Calcium flux assay were performed using either INDO-1AM or Fluo-3am as probe, as indicated.

INDO-1AM. Lymph nodes were made into single-cell suspensions and FLT3L-generated dendritic cells were incubated in 2 μM final concentration of INDO-1AM (BD Biosciences) in RPMI supplemented with 50 μM β-mercaptoethanol, and 1% FBS for 30 min in a 37 °C, 5% CO_2_ humidified incubator. Lymphocytes were washed and spun at 1,300 rpm for 5 min followed by cell surface staining for CD4^+^ (clone GK1.5), CD8^+^ (clone 2.43), B220 (clone RA3-6B2; Invitrogen eBioscience) and viable cells were distinguished using the Fixable Viability Dye 780 (Invitrogen, eBioscience) for 15 min. Cell suspensions were spun and resuspend in the aforementioned media at 3 × 10^6^ cells per ml and kept on ice. cDC cultures were stained for CD11c (clone N418), B220 (clone RA3-6B2), MHCII (clone AF700), SIRPΑ1 (clone p84) and XCR1 (clone ZET). Cells were heated to 37 °C for 5 min before acquisition on the LSR Fortessa (BD Biosciences). Anti-CD3–biotin (5 μg ml^−1^) (clone 145-2C11; Invitrogen eBioscience) was added during baseline reading for 1–2 min, followed by 20 μg ml^−1^ of streptavidin (Invitrogen). For B cells, 20 μg ml^−1^ anti-IgM (Jackson ImmunoResearch, 115-006-020) was injected into the tube after baseline reading. Dendritic cell samples were injected with 1 mM ATP after baseline reading. Calcium flux was determined by the ratio between 400 nm (bound) to 500 nm (free) readings. Samples were acquired on the BD LSR Fortessa. Flow cytometry data was analysed using FlowJo v10 (TreeStar). To quantify the percentage of responders, cells were gated after the addition of streptavidin for 250 s.

FLUO-3AM. Single-cell suspensions were obtained from lymph nodes. Cells were loaded with the calcium indicator Fluo-3AM (Abcam) as follows: cells resuspended in TCM in presence or absence of sucralose were incubated for 30 min with 5 μM Fluo-3 AM diluted 1:1 (v/v) in 20% (w/v) Pluorinic F-127 acid (Merck, P2443) at 37 °C followed by a further incubation of 15 min at room temperature. Excess Fluo-3AM was removed by two consecutive washes with cold calcium-free PBS. Cells were finally resuspended in calcium-free PBS supplemented with 10 mM glucose, 2 mM glutamine and 50 μM β-mercaptoethanol and kept on ice. Cells were warmed to 37 °C for 5 min before acquisition using the LSR Fortessa. Baseline reading were recorded for 1–2 min followed by stimulation of intracellular calcium release with either 1 μM thapsigargin (Merck) or 100 ng ml^−1^ ionomycin (Merck). For quantification, Fluo3 intensity was plotted against time using FlowJo (TreeStar) kinetic option with the mean value and Gaussian smoothing. A range of the same length of time was then selected for both basal and treatment (thapsigargin or ionomycin) condition and the area under the curve was obtained.

### Jurkat T cell proliferation and sucralose wash-off in vitro experiment

Jurkat T Cells were cultured in RPMI supplemented with 10% FBS, 50 μM β-mercaptoethanol and 1% penicillin-streptomycin. Proliferation was measured by counting cells with a CASY counter in presence of the different sweeteners as indicated.

Sucralose wash-off in vitro experiment. For acute sucralose exposure, Jurkat T cells were cultured in presence or absence of 0.5 mM sucralose for 5 days. For chronic exposure: sucralose on/on and sucralose on/off Jurkat T cells were pre-exposed to 0.5 mM sucralose for 2 weeks before sucralose removal (sucralose on/off) or continuous sucralose exposure (sucralose on/on).

### Membrane order measurement

T cells isolated from C57BL/6J male mice aged between 6 and 10 weeks—were activated with anti-CD3 (2 μg ml^−1^) and anti-CD28 (1 μg ml^−1^) for 3 days. Cells were then stained for protein surface markers and viability dye before being loaded with the phase sensitive membrane probe 2 μM Di-4-ANEPPDHQ (ANEq, Thermo Fisher, D36802) in RPMI in the presence of 0.02% Pluronic F-127 (Merck, P2443). The loading was performed for 30 min at 37 °C followed by 15 min at room temperature. Cells were kept at 37 °C and protected from light before acquisition. ANEq fluorescence emission was measured at 570 nm (high order) and 610 nm (low order) using a BD LSR Fortessa. Flow cytometry data was analysed using FlowJo v10 (TreeStar).

### Immunoblotting

Cells were lysed using RIPA buffer (Millipore) supplemented with 1% SDS and phosphatase and protease inhibitor cocktail (La Roche), denatured at 95 °C, and resolved on NuPAGE polyacrylamide pre-cast gels (Invitrogen, Thermo Fisher Scientific). Gels were transferred onto nitrocellulose membranes (GE Healthcare). T cell or Jurkat T cell lysates were probed as indicated in the manuscript. Working dilution of primary and secondary antibodies are listed in the antibodies section. All uncropped and unprocessed scans and images are in the Source Data files.

### Immunoprecipitation

Jurkat T cells were cultured in RPMI supplemented with 10% FBS, 50 μM β-mercaptoethanol and 1% penicillin-streptomycin in the presence or absence of 0.5 mM sucralose for 48 h. Cells were washed once with PBS and resuspended at the concentration of 30 × 10^6^ cells ml^–1^ in DPBS containing 1% FBS, 5 mM glucose, 2 mM glutamine, 50 μM β-mercaptoethanol and 1% penicillin-streptomycin in the presence or absence of 0.5 mM sucralose.

Cells were kept on ice and activated with 5 μg ml^−1^ anti-CD3 (OKT3) at 37 °C as indicated. The reaction was stopped by adding ice-cold PBS. Proteins were extracted using immunoprecipitation lysis buffer containing 0.2% Triton X-100, 50 mM Tris-HCl pH 7.5, 150 mM NaCl, protease inhibitor cocktail (Thermo Scientific) and phosphatase inhibitor cocktail (Cell Signaling) at 4 °C for 30 min. Total protein content was quantified using a BCA assay. CD3ζ was immunoprecipitated from 1.5 mg total protein using an anti-CD3ζ antibody (Santa Cruz), using 4 μg antibody per 0.5 mg total protein and Protein A/G plus Agarose (Thermo Fisher) for 2 h at 4 °C. Immunoprecipitated proteins were washed three times with immunoprecipitation lysis buffer and western blots were performed as indicated.

### Tumour digestion and plasma collection

#### Tumours

Excized tumours were kept in ice-cold medium until processing. Tumours were chopped into 1-mm pieces and digested in digestion buffer containing 0.012% collagenase, 0.012% dispase, 0.1 mg ml^−1^ DNase I, 1% FBS in Krebs Ringer bicarbonate buffer (KRB) for 45–60 min at 37 °C with gentle oscillation. Cold DMEM supplemented with 10% FBS (10 ml) was used to neutralize the digestion. Single cells were filtered through 100-μm cell strainer and collected by centrifugation at 300*g* for 5 min.

#### Plasma

Blood was collected by cardiac puncture into EDTA-coated tubes. Blood was spun in 1.5 ml Eppendorf tubes at 2,000*g* for 15 min at 4 °C. The supernatant (plasma) was collected and stored at −80 °C.

### Enzyme linked immunosorbent assay

ELISAs were performed on T cell supernatants for IL-2 (Invitrogen, eBioscience) and serum from LPS-challenged mice was assessed for circulating IL-1β levels (Sigma). ELISAs were performed following the manufacturer’s procedures.

#### Insulin

Blood was collected by cardiac puncture in EDTA-coated tubes. Samples were then centrifuged at 2,000*g* for 15 min to remove red blood cells and plasma insulin was measured using Insulin ELISA KIT (Alpco, 80-INSMS-E01) per the manufacturer’s instructions.

### RNA isolation and RNA-seq

CD4^+^ T cells were isolated from lymph nodes and spleen from male C57BL/6J mice using CD4^+^ T cell negative isolation kits (StemCell Technologies) according to the manufacturer’s protocol. 2 × 10^6^ CD4^+^ T cells per well were plated in 24-well plates precoated overnight with 5 μg ml^−1^ anti-CD3 and 2 μg ml^−1^ anti-CD28 treated with various sweeteners (0.5 mM of sweetener). T cells were collected 24 h and 48 h after plating and total RNA was isolated using TriPure Isolation Reagent (Merck) according to manufacturer’s instructions. RNA was quantified using a nanodrop (deNovix).

mRNA capture and library preparation were performed by the Advanced Sequencing Facility at the Francis Crick Institute using the KAPA mRNA HyperPrep Kit (Roche). Technical triplicate libraries were sequenced on an Illumina HiSeq 4000 platform to generate on average 50 million 101-bp single-end reads per sample.

Raw reads were quality and adapter trimmed using cutadapt (version 2.10) before alignment^50^. Reads were mapped and subsequent gene-level counted using RSEM 1.3.1 (ref. ^51^) and STAR 2.7.6 (ref. ^52^) against the mouse genome GRCm38 using annotation release 95, both from Ensembl. Normalization of raw count data and differential expression analysis was performed with the DESeq2 package (version 1.30.1)^47^ within the R programming environment (v4.0.3)^45^. The following pairwise comparisons were performed: sucralose 24 h and TCM 24 h; sucralose 48 h and TCM 48 h; saccharin 24 h and TCM 24 h; saccharin 48 h and TCM 48 h; sucralose 24 h and saccharin 24 h; sucralose 48 h and saccharin 48 h; sucralose 48 h and sucralose 24 h; saccharin 48 h and saccharin 24 h; TCM 48 h and TCM 24 h, with the contrast function, from which genes differentially expressed (adjusted *P* value being less than 0.01) between different conditions were determined. Gene lists were used to look for pathways and molecular functions with over-representation analysis using DAVID^53,54^.

### Sucralose quantification and stable isotope tracing by LC–MS

Stable isotope tracing of U-[^13^C]glucose (Cambridge Isotopes) was performed in glucose-free IMDM (The Francis Crick Media Services) supplemented with 10% dFBS. CD4^+^ and CD8^+^ T cells were activated with anti-CD3 (5 mg ml^−1^) and anti-CD28 (2 mg ml^−1^) for 48 h in the presence and absence of 0.5 mM sucralose. T cells were counted, washed, and replaced with the 10 mM U-[^13^C]glucose solution at a concentration of 2 × 10^6^ cells ml^−1^ followed by a 4 h pulse. Cells were washed twice with ice cold PBS and metabolites were extracted using extraction buffer containing 50% methanol, 30% acetonitrile and 20% water for 10 min at 4 °C. For sucralose detection in Jurkat T cells, cells were exposed to 0.5 mM sucralose for 48 h unless otherwise indicated, and washed 2 times with ice cold PBS. Metabolites were extracted as described above using 1 ml of extraction buffer for 3.5 × 10^6^ cells. For sucralose detection in plasma, the extraction procedure was adapted from ref. ^55^. In brief, metabolites were extracted as follows: plasma samples were allowed to thaw on ice for 30–60 min. Ice-cold methanol was subsequently added to 50 µl of plasma in a ratio 3:1 (v/v). After a short vortex step, the mixture was incubated on ice for 5 min. Centrifugation (13,000 rpm, 10 min, 4 °C) was used to pellet the protein. The supernatant was transferred to an Eppendorf tube and dried in a rotary vacuum concentrator. Polar metabolites were phase-partitioned from apolar metabolites by addition of 350 μl chloroform:methanol:water (1:3:3 v/v/v, containing 0.375 mol of [^13^C]valine as an internal standard). The phases were separated by centrifugation (13,000 rpm, 10 min, 4 °C). The polar phase was transferred into a LC–MS vial equipped with an insert and dried in a rotary vacuum concentrator. Finally, 75 µl of a mixture of H_2_O:methanol (1:1) were added to the dried extract. The samples were subsequently analysed by LC–MS. For the preparation of the sucralose calibration curve, 50 µl of plasma was spiked with various concentrations of the standard. The extraction and sample preparation were then performed as described above.

Metabolites and sucralose were analysed by LC–MS using a Q-EXACTIVE Plus (Orbitrap) mass spectrometer from Thermo Fisher Scientific coupled with a Vanquish UHPLC system from Thermo Fisher Scientific. The chromatographic separation was performed on a SeQuant ZicpHILIC (Merck Millipore) column (5 μm particle size, polymeric, 150 × 4.6 mm). The injection volume was 5 μl, the oven temperature was maintained at 25 °C, and the autosampler tray temperature was maintained at 4 °C. Chromatographic separation was achieved using a gradient program at a constant flow rate of 300 μl min^−1^ over a total run time of 25 min. The elution gradient was programmed as decreasing percentage of B from 80% to 5% for 17 min, holding at 5% of B for 3 min and finally re-equilibrating the column at 80% of B for 4 min. Solvent A was 20 mM ammonium carbonate solution in water supplemented by 1.4 ml l^−1^ of a solution of ammonium hydroxide at 35% in water and solvent B was acetonitrile. Mass spectrometry was performed with positive/negative polarity switching using a Q-EXACTIVE Plus Orbitrap (Thermo Scientific) with a HESI II probe. Mass spectrometry parameters were as follows: spray voltage 3.5 and 3.2 kV for positive and negative modes, respectively; probe temperature 320 °C; sheath and auxiliary gases were 30 and 5 arbitrary units, respectively; and full scan range: 70–1,050 *m*/*z* with settings of AGC target and resolution as balanced and high (3 × 10^6^ and 70,000), respectively. Data were recorded using Xcalibur 4.2.47 software (Thermo Scientific). Mass calibration was performed for both ESI polarities before analysis using the standard Thermo Scientific Calmix solution. To enhance calibration stability, lock-mass correction was also applied to each analytical run using ubiquitous low-mass contaminants. Parallel reaction monitoring (PRM) acquisition parameters were the following: resolution 17,500; collision energies were set individually in HCD (high-energy collisional dissociation) mode. Metabolites were identified and quantified by accurate mass and retention time and by comparison to the retention times, mass spectra, and responses of known amounts of authentic standards using TraceFinder 4.1 EFS software (Thermo Fisher Scientific). Absolute quantification of sucralose was achieved with the use of a calibration curve built with the responses of known amounts of standard spiked in sucralose-free plasma.

### Subcellular fractionation

Cell fractions were obtained as described previously^56^ with minor modifications as reported below.

#### Metabolomics

Jurkat T cells were cultured with or without 0.5 mM sucralose for 48 h. In total, 3.5 × 10^6^ cells were washed twice with ice-cold PBS then digested with 1 ml PBS containing 0.5 mg ml^−1^ digitonin (BioVision) to release cytoplasmic content, followed by centrifugation for 10 s at 13,500*g*. Two-hundred microlitres of the supernatant, containing the cytoplasmic fraction, was collected in a new, pre-chilled tube containing 800 µl of ice-cold extraction buffer. The pellet was resuspended in 100 μl of ice-cold extraction buffer. The metabolites were extracted and processed as described above. For whole cells controls, cells (3.5 × 10^6^) cultured in presence or absence of 0.5 mM sucralose were washed twice with ice-cold PBS and extracted in 1 ml ice-cold extraction buffer.

#### Western blot

Jurkat T cells were cultured with or without 0.5 mM sucralose for 48 h. Cells (3.5 × 10^6^) were washed once with ice-cold PBS then digested with 1 ml PBS containing 0.5 mg ml^−1^ digitonin to release cytoplasmic content followed by centrifugation for 10 s at 13,500*g*. The supernatant containing the cytoplasmic fraction was collected in a new, pre-chilled tube. The pellet was resuspended in 1 ml ice-cold PBS. Cells (3.5 × 10^6^) untreated with digitonin were used as whole-cell controls. All the samples were stored at −80 °C for at least 12 h and sonicated 3 times for 10 s on ice. The purity of the fraction was tested using specific antibodies as indicated.

### Sucralose localization by cryo-OrbiSIMS

Primary or Jurkat T cells were treated for 48 h with 0.5 mM deuterium-labelled sucralose (SCBT). Sucralose localization was performed by cryo-OrbiSIMS analysis using a Hybrid-SIMS instrument (IONTOF GmbH, Thermo Fisher Scientific) at the National Physical Laboratory incorporating an Orbitrap Q-Exactive HF analyser and a time of flight (ToF) analyser^57, 58^. T cells were deposited onto silicon wafers coated with 50 nm gold, excess media removed, and frozen by plunging into liquid nitrogen. The samples were transported under liquid nitrogen and mounted on a custom Leica sample holder under liquid nitrogen in a Leica VCM and transferred into the OrbiSIMS onto a sample stage pre-cooled to −160 °C. For charge compensation, a 21 eV electron flood gun was used with a current of −21 μA, and argon gas flooding in the analysis chamber with a pressure of 9.7 × 10^−7^ mbar. All acquisitions were performed in positive polarity with an extraction voltage of 2 kV and a sample potential of ~−80 V. 3D analysis was performed using the ToF analyser with a cycle time of 200 μs. A 30 keV Bi^+^ liquid metal ion gun (LMIG) with a spot size of ~500 nm was used as the analysis beam with a current of 1.74 pA, and a 10 keV Ar^2081+^ gas cluster ion beam (GCIB) was used as the sputter beam with a current of 0.618 nA. The 3D image was acquired using 10 shots per pixel per frame and 3 frames per scan with a field of view of 200 μm × 200 μm in sawtooth raster mode, with non-interlaced sputtering of 440 s between frames with a crater size of 500 μm × 500 μm. Analysis was performed at a temperature of ~−160 °C throughout. The instrument liquid nitrogen dewars were loosely covered with polyethylene bags to prevent excessive ice formation. The *m*/*z* scale of the mass spectrum was calibrated using the following peaks: CH^+^, CH^2+^, CH^3+^ and Na^+^. Sucralose peaks were identified compared with analysis of a pure standard and confirmed by isotope cluster distribution. Spectral analysis was performed using the Orbitrap analyser with a cycle time of 200 μs. A 20 keV Ar^3500+^ GCIB was used as the analysis beam with a current of 172 pA, a duty cycle of 30% and a spot size of ~20 μm. The analysis was performed using a 40 μm × 40 μm field of view and a 20 μm pixel size with random raster mode. The collisional cooling He pressure was 3.9 × 10^−2^ mbar. The mass range was 150–600 *m*/*z* and the injection time was 2,000 ms at a mass resolution of 240,000 Δ*m*/*m* with a transient time of 512 ms. Mass calibration was performed using silver clusters generated from a silver sample. Data were acquired and analysed using SurfaceLab 7.3 (IONTOF GmbH). For the depth profile shown in Fig. 2d, relative increasing depth is indicated by consecutive data points. After each data point, the GCIB sputter removes material. The grey region in Fig. 2d indicates the region below which the ion signal is considered to be noise, this value is calculated from an untreated control sample. The data represents the mean and s.e.m. of eight separate cells located within the same field of view normalized to total ion count. The cell regions of interest were selected using the ion [C_2_HO]^+^ as a cell-specific marker.

### Reporting summary

Further information on research design is available in the Nature Portfolio Reporting Summary linked to this article.

## Online content

Any methods, additional references, Nature Portfolio reporting summaries, source data, extended data, supplementary information, acknowledgements, peer review information; details of author contributions and competing interests; and statements of data and code availability are available at 10.1038/s41586-023-05801-6.

## Supplementary information

Supplementary Information This file contains Supplementary Figs. 1–4 and a list of reagents.

Reporting Summary

Supplementary Fig. 1 Uncropped western blots

## Data Availability

The dataset generated for the gut microbiome and T cell RNA-seq are available at GEO (GSE220862) and SRA (PRJNA913879). Source data are provided with this paper.

## Source data

Source Data Fig. 1
Source Data Fig. 2
Source Data Fig. 3
Source Data Fig. 4
Source Data Extended Data Fig. 1
Source Data Extended Data Fig. 2
Source Data Extended Data Fig. 3
Source Data Extended Data Fig. 4
Source Data Extended Data Fig. 5
Source Data Extended Data Fig. 6
Source Data Extended Data Fig. 7
Source Data Extended Data Fig. 8
Source Data Extended Data Fig. 9

## Code availability

All R scripts necessary to reproduce the metagenomic analysis are available at 10.5281/zenodo.7448236. All R scripts necessary to reproduce the RNA-seq analysis are available at https://zenodo.org/record/7469893#.Y6NPaHbP2Uk.
