# Supplementary material for: The dietary sweetener sucralose is a negative modulator of T cell-mediated responses
Source: Nature. 2023 Mar 15;615(7953):705–11. doi: 10.1038/s41586-023-05801-6 (PMC10033444; doi:10.1038/s41586-023-05801-6)
Supplement: Supplementary file 1 — This file contains Supplementary Figs. 1–4 and a list of reagents. [file 41586_2023_5801_MOESM1_ESM.docx]

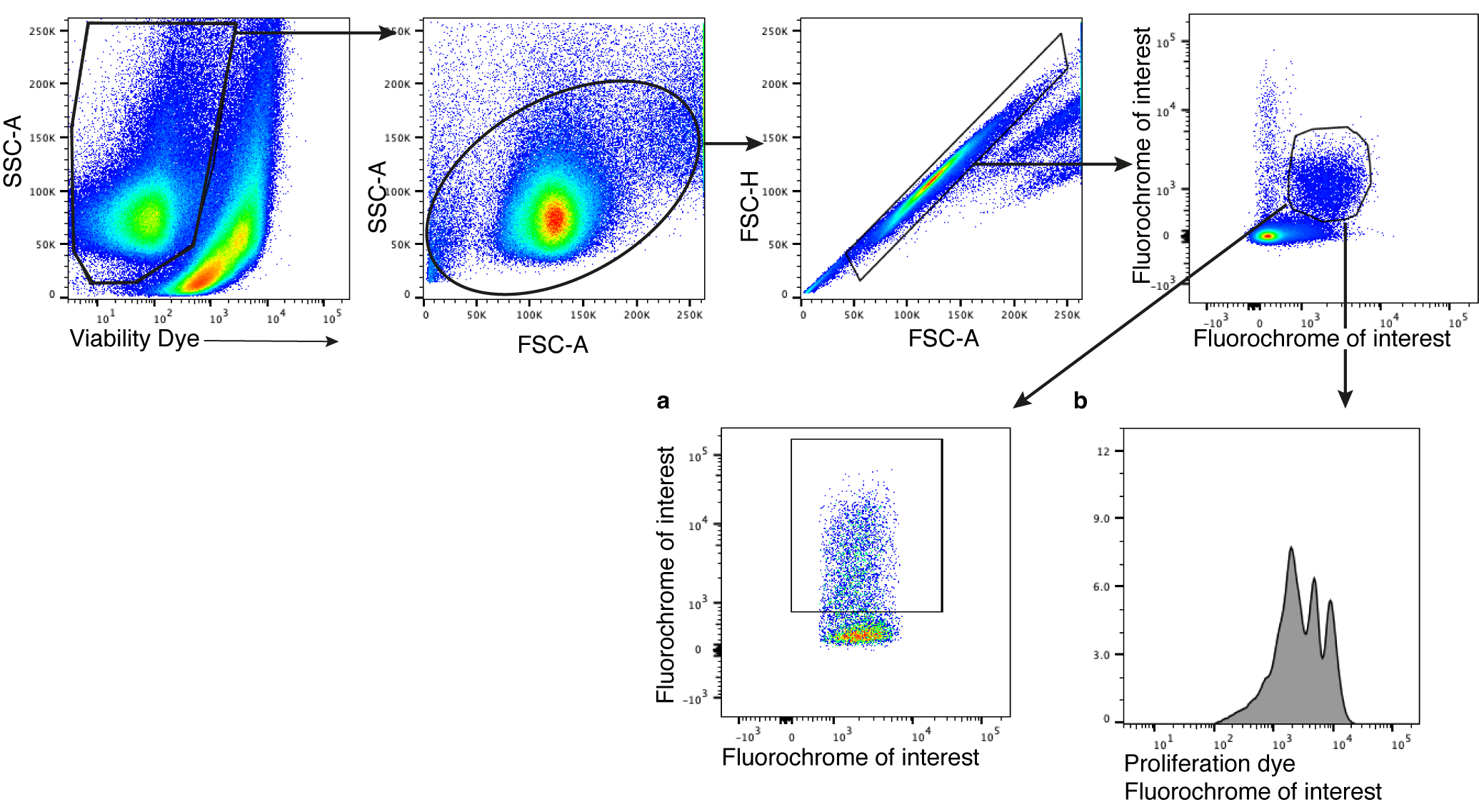


**Supplementary Information Figure 1- Representative Flow Cytometry Gating Strategy**

Representative gating strategy followed for flow cytometry experiments. Dead cells were stained with the Fixable Viability Dye eFluoro^TM^ 780 or similar dyes and excluded. Doublets were excluded using forward scatter (FSC) area versus height.

**a)** ***Gating strategy for***: Figure 1h-k; Figure 2l and m; Figure 3a-c, i-k, n-p; Figure 4d-h. Extended Data 1b-g, i-k, Extended Data 7g; Extended data 8a-d, f-h, and k; Extended Data 9d-g, and i.

**b)** ***Gating strategy for:*** Figure 1 d-g; Extended Data 2a and b; Extended Data 5a, f, g, h, and k; Extended Data 6a; Extended Data 7f.

**Supplementary Information Figure 2- Representative Flow Cytometry Gating Strategy**

Representative gating strategy followed for flow cytometry experiments. Dead cells were stained with the Fixable Viability Dye eFluoro^TM^ 780 and excluded. Doublets were excluded using forward scatter (FSC) area versus height. Calcium flux was derived between the 406/485 emission ratios (termed derived) and measured over time.

***Gating strategy for***: Figure 2h-k; Extended Data 7a, j-k.


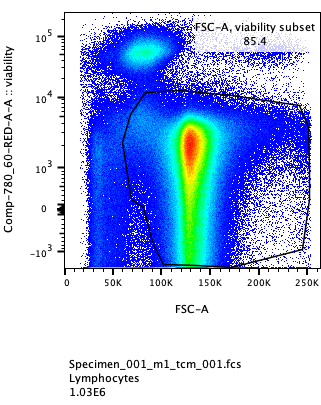

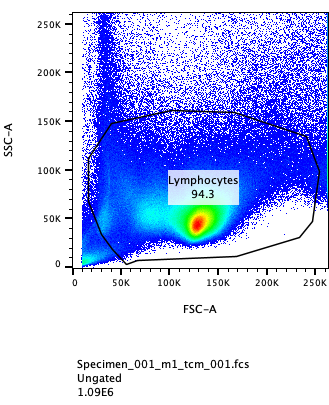

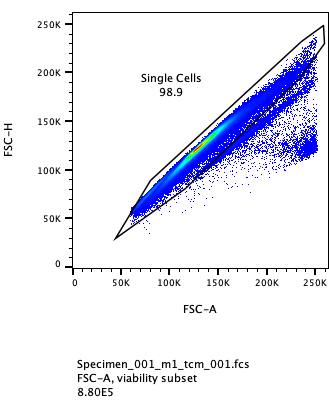

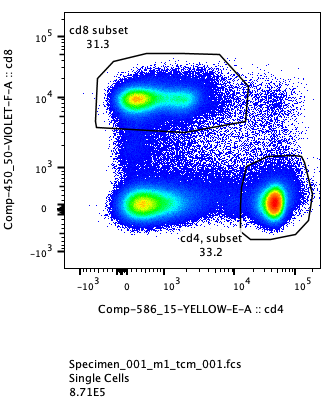

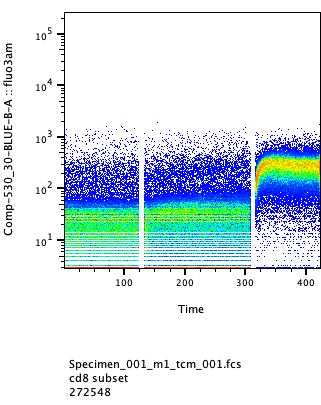

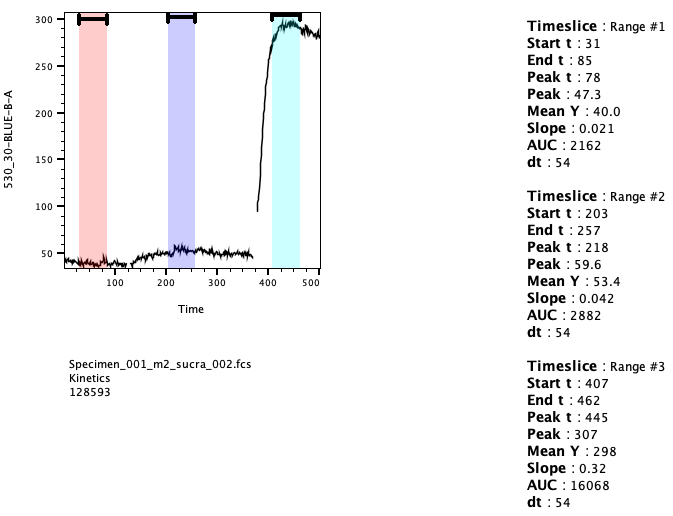


SSC-A

FSC-A

Viability Dye

FSC-A

FSC-A

FSC-H

Fluorochrome of interest

Fluorochrome of interest

Fluorochrome of interest

Time

Time

Mean Fluor. of interest

**Supplementary Information Figure 3- Representative Flow Cytometry Gating Strategy**

Representative gating strategy followed for flow cytometry experiments. Lymphocytes were gated using FSC-A vs SSC-A. Dead cells were stained with the Fixable Viability Dye eFluoro^TM^ 780 and excluded. Doublets were excluded using forward scatter (FSC) area versus height. CD4 and CD8 cells were separated using different Fluorochromes. Calcium flux was measured using emission 506/526 measured over time.

***Gating strategy for***: Extended Data 2d; Extended Data 4h; Extended data 7b-e.


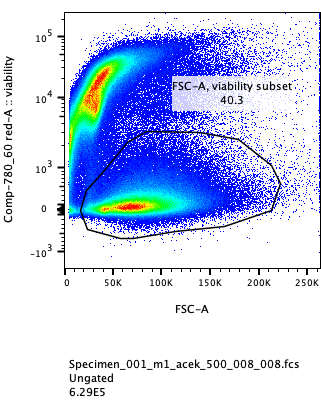

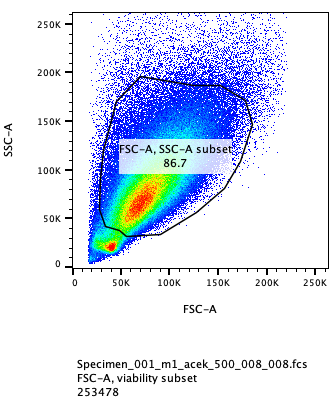

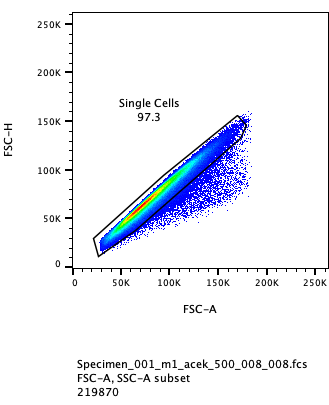

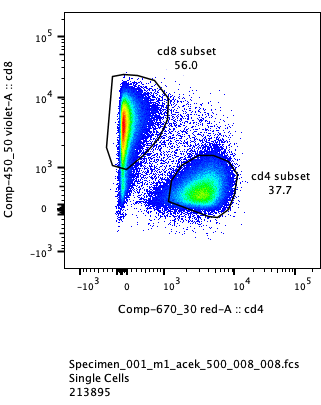

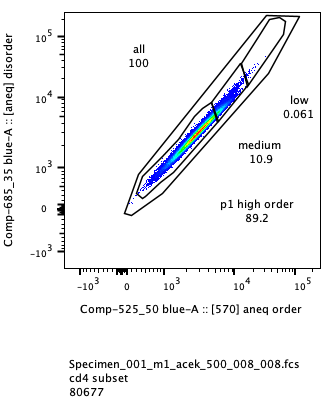


SSC-A

FSC-A

FSC-A

Viability Dye

FSC-A

FSC-H

Fluorochrome of interest

Fluorochrome of interest

Fluorochrome of interest

Fluorochrome of interest

**Supplementary Information Figure 4- Representative Flow Cytometry Gating Strategy**

Representative gating strategy followed for flow cytometry experiments. Dead cells were stained with the Fixable Viability Dye eFluoro^TM^ 780 and excluded. Lymphocytes were gated using FSC-A vs SSC-A. Doublets were excluded using forward scatter (FSC) area versus height. CD4 and CD8 cells were separated using different Fluorochromes. Membrane order was measured using emission at 525/50 (high order) vs 685/30 (low order)

***Gating strategy for***: Figure 2e; Extended Data 6 h, i.

**Supplementary Information List-1: List of Antibodies**

**Cell Signalling**

Phospho-PLC gamma 1 Tyr783 (CST, 14008; clone D6M9S; Lot 4; 1:1000)

Phospho-ZAP70 Tyr319 (CST,2717, clone 65e4; lot:15; 1:500)

Phospho-Lck Tyr505 (CST; 2751; Lot 1; 1:1000)

Phospho-LAT Tyr220 (CST; 3584; Lot 1; 1:1000)

Phospho-STAT5(Y694) (CST 4322P; clone D47E7; lot 4; 1:1000)

Phospho-ERK1/2 (p44/42 MAPK) (CST; 9101; Lot 30; 1:1000)

CD3 zeta (CST 88083, Lot:1. 1:1000)

Lck (CST; 2984, Clone CD88; Lot:1, 1:1000)

ZAP70 (CST; 2705, clone 99F2; lot:10, 1:1000)

Anti-rabbit IgG HRP-Linked (CST 7074 Lot 25; 1:5000)

NaK ATPase (CST; 3010S, lot:5. 1:1000)

Tom20 (CST; 42406s, D8T4N; lot 1; 1:1000)

β-actin (CST 4970; clone I3E5; lot 1:1000)

**Abcam**

β-actin (Abcam ab8229; lot gr315283-22; 1:1000)

Alexa488 Goat pAb to Ms IgG (Abcam ab150113; GR3353661-2; 1:1000)

**LiCOR**

IRDye®800CW Donkey anti-mouse (926-32212, Lot C9110232-09, 1:20000)

IRDye®680CW Donkey anti-mouse (926-68022, Lot D00128-03, 1:20000)

IRDye®680LT Donkey anti-Rabbit (926-68023, Lot D10209-15, 1:20000)

RDye®800CW Donkey anti-Rabbit (926-32213, Lot C90806-09, 1:20000)

**Invitrogen (eBioscience)**

FITC IL-1b-pro (eBioscience; 11-7114-82 clone NJTEN3; lot 234982; 1:250)

FITC-CD4 (eBioscience; 11-0042-85; Clone RM4-5; 2057560; 1:300)

FITC MHC Class II (I-A/I-E) (clone M5/114.15.2; 11-5321-82)

PE-B220 (eBioscience; 12-0452-83; clone RA3-6B2; Lot 4290694; 1:300)

PE-CD44 (eBiosceince; 120441-81; clone IM7; Lot 3389547; 1:300)

PE-Cy7-CD4 (eBioscience; 25-0041-82; clone GK1.5; Lot 1993630; 1:300)

PE-Cy7-Granzyme B (eBioscience; 25-8898-80; clone NGZB; Lot 1986381; 1:250)

PE-Relm alpha 1 (eBioscience; 12-5441-82; clone DS8RELM; Lot 2356244; 1:300)

PE-FOXP3 (eBioscience; 12-5773-82; clone FJK-16s; Lot 2144983; 1:250)

APC-CD25 (eBiocience; 11-0251-82; clone PC61.5; lot 2018381; 1:300)

APC-NK1.1 (eBioscience; 17-5941-82; clone PK136; lot 1998276; 1:300)

APC-780-CD11b (eBioscience; 47-0112-82; clone M1-70; lot 4290718; 1:300)

eFluor450-TCRb (eBioscience; 48-5961-82; clone H57-597; lot 4335198; 1:300)

eFluor450-CD44 (eBioscience; 48-0441-82; clone IM7; lot 1983649; 1:300)

Biotin-anti-CD3e (eBioscience; 130031-85; clone 145-2C11; 1:200)

eFluor450-CD8a (eBioscience; 48-0081-82; clone 53-6.7; lot 2093800; 1:300)

eFluor450-CD11c (eBioscience; 48-0114-80; clone N418; lot 1994180)

anti-Mouse CD28 functional grade (clone 37.51; 16-0281-86; 1:500)

anti-Mouse CD3e functional grade (clone 145-2C11; 16-0031-86; 1:200)

anti-Human CD3e (clone OKT3; 2373795; 1:100)

**TONBO Bioscience**

FITC-IFNg (TONBO; 35-7311; clone XMG1.2; C7311092719353; 1:300)

Violet Fluor450-CD4 (TONBO; 750042; clone RM4-5; C0042081618753; 1:300)

PerCP-cyanine5.5-CD4 (TONBO; 65-0041-u100; clone CK1.5; C0041082619653; 1:300)

**BioLegend**

FITC-Annexin

FITC-F4/80 (Biolegend; 123107; clone BM8; LOT B287205; 1:300)

PE-CD4 (Biolegend; 100408; clone GK1.5; LOT B248731; 1:300)

PE-CD8a (Biolegend; 100708; clone 53-6.7; LOT B268831; 1:300)

PE XCR1 (Biolegend; 148203; clone ZET; Lot B285069; 1:200)

PE-TCR beta chain (Biolegend; 109207; clone H57-597; LOT B308457)

PerCP-Cy5.5 B220 (clone RA3-6B2; 103236; Lot B285069; 1:200)

PerCP-Cy5.5 Ki67 (Biolegend; 652424; clone16A8; lot b235166; 1:250)

PE-Cy7-Tbet (Biolegend; 644824; clone 4B10; lot b255602; 1:250).

PE-Cy7-CD206 (Biolegend; 141719; clone C068C2; lot b318357; 1:300)

APC-TNF (Biolegend; 506308; clone MP6-XT22; lot b255367; 1:250)

APC-GL7 (Biolegend; 144618; clone GL7; lot b287646; 1:300)

APC-CD45RB (Biolegend; 103319; clone C363-16A; lot 2082880; 1:300)

APC-CD301 (Biolegend; 145707; clone LOM-14; lot b312436; 1:300)

Alexa Fluor 647 Sirp alpha (Biolegend; 144028; clone p84; Lot B272850; 1:200)

BV421-Tbet (Biolegend; 644815; clone 4B10; lot b317403; 1:250)

BV605-CD95 (Biolegend; 152612; clone SA367H8; lot b261359; 1:300)

BV650-CD69 (Biolegend; 104541); clone H1.2F3; lot b258598; 1:300)

BV650 CD11c (Biolegend; 117339; clone N418; Lot B349622; 1:200)

Anti-mouse IL4 (clone 11B11; 504102)

FITC-CD8a (Biolegend; 100706; clone53-6.7; lot b298556; 1:300)

BV711-CD4 (Biolegend; 100557; clone RM4-5; lot b35831; 1:300)

FITC-NK1.1 (Biolegend; 108706; clone PK136; lot b236605; 1:300)

BV785-B220 (Biolegend; 103246; clone RA3-6B2; lot b256836; 1:300)

PeCy7-CD8 alpha (Biolegend; 100722; clone 53-6.7; lot b357207; 1:300)

**BD-Pharmigen**

PE-Cy7-CD45.1 (BD; 560578; clone A20; LOT 0307154; 1:300)

PE-CD184 (BD; 551955; Lot 1204181; 1:300)

**Baylor College of Medicine - MHC Tetramer Facility**

PE-Kb/OVA257 (19027)

**Santa Cruz (SC)**

PLCg1 (SC; sc7290; clone E-12, lot: H1518; 1:1000)

STAT5 (SC; sc74442; clone A-9; LOT F1421; 1:1000)

GAPDH (SC; sc-322333, Clone D0722, lot; 1:1000)

ZAP70 (SC; sc-32760; clone 1E7.2; LOT D1217; 1:1000)

Lck (SC; sc-433; clone 3A5; LOT C2519; 1:1000)

LAT (SC; sc-53550; clone 11b.12; LOT C1722; 1:500)

ERK1/2 (SC; sc-514302; clone C9; LOT C1722; 1:1000)

CD3 zeta (clone 6B10.2; sc-1239, used for IP only as indicated)

Mouse IgG (sc-2025, Lot2721, used for IP only)
