# Supplementary material for: The dietary sweetener sucralose is a negative modulator of T cell-mediated responses
Source: Nature. 2023 Mar 15;615(7953):705–11. doi: 10.1038/s41586-023-05801-6 (PMC10033444; doi:10.1038/s41586-023-05801-6)
Supplement: Supplementary file 2 — Reporting Summary [file 41586_2023_5801_MOESM2_ESM.pdf]

nature research

Corresponding author(s): KH Vousden, J Blagih, F Zani

Last updated by author(s): December 19, 2022

Reporting Summary

Nature Research wishes to improve the reproducibility of the work that we publish. This form provides structure for consistency and transparency in reporting. For further information on Nature Research policies, see our Editorial Policies and the Editorial Policy Checklist.

Statistics

For all statistical analyses, confirm that the following items are present in the figure legend, table legend, main text, or Methods section.

n/a	Confirmed	
☐	☒	The exact sample size (*n*) for each experimental group/condition, given as a discrete number and unit of measurement
☐	☒	A statement on whether measurements were taken from distinct samples or whether the same sample was measured repeatedly
☐	☒	The statistical test(s) used AND whether they are one- or two-sided<br>*Only common tests should be described solely by name; describe more complex techniques in the Methods section.*
☒	☐	A description of all covariates tested
☐	☒	A description of any assumptions or corrections, such as tests of normality and adjustment for multiple comparisons
☐	☒	A full description of the statistical parameters including central tendency (e.g. means) or other basic estimates (e.g. regression coefficient) AND variation (e.g. standard deviation) or associated estimates of uncertainty (e.g. confidence intervals)
☐	☒	For null hypothesis testing, the test statistic (e.g. *F*, *t*, *r*) with confidence intervals, effect sizes, degrees of freedom and *P* value noted<br>*Give P values as exact values whenever suitable.*
☒	☐	For Bayesian analysis, information on the choice of priors and Markov chain Monte Carlo settings
☒	☐	For hierarchical and complex designs, identification of the appropriate level for tests and full reporting of outcomes
☒	☐	Estimates of effect sizes (e.g. Cohen's *d*, Pearson's *r*), indicating how they were calculated

Our web collection on statistics for biologists contains articles on many of the points above.

Software and code

Policy information about availability of computer code

Data collection	Flow cytometry data was collected using the BD FACSDiva software V 8.0.1. Western blot fluorescence analysis was collected using the LiCor Image Studio Software V 5.2. Absorbency data was collected using Tecan Safire2 (magellan V 7.2). Indirect calorimetry data were collected using PhenoMaster software (TSE systems)Version: 7.0.7.7250 . body composition was measured using a Echo-MRI device (Echo-MRI) E56-073-MTB. Images were taken on a Zeiss Upright 710 using the ZEN (v2.3) program.
Data analysis	GraphPad (Prism) V9.4.0, Mass spectrometry data: ChemStation E.02.02.1431, Immunoblot : Fiji ImageJ V 2.0.1, Flow cytometry data: FlowJo V 10.8.2, Thermo TraceFinder. RNA-seq analysis; Trimming – cutadapt (version 2.10), Mapping – RSEM (1.3.1) and STAR (2.7.6). Mapped to gene against the mouse genome GRCm38 using annotation release 95– Differential gene expression – Deseq2 (Bioconductor) within R programming environment (version 4.0.3). for the gut microbiome analysis The fastq files were processed using DADA2 v 1.18; Taxa and species assignment was carried out using v132 of the silva database. The processed data were then analysed in R (4.0.334) with the phyloseq package35 aggregating the count data to the genus level. DESeq2 (v1.30 and 1.30.1) was used to estimate the log-fold changes and p-values between experimental groups. for the cryo-OrbiSIMS data were analysed using SurfaceLab version 7.3 (IONTOF).

For manuscripts utilizing custom algorithms or software that are central to the research but not yet described in published literature, software must be made available to editors and reviewers. We strongly encourage code deposition in a community repository (e.g. GitHub). See the Nature Research guidelines for submitting code & software for further information.

Data

Policy information about availability of data

All manuscripts must include a data availability statement. This statement should provide the following information, where applicable:

- Accession codes, unique identifiers, or web links for publicly available datasets
- A list of figures that have associated raw data
- A description of any restrictions on data availability

All data are available upon reasonable request and can be found within the manuscript and supplementary information. RNA-seq and gut microbiome data has been deposited and made publicly available.

Field-specific reporting

Please select the one below that is the best fit for your research. If you are not sure, read the appropriate sections before making your selection.

☒ Life sciences ☐ Behavioural & social sciences ☐ Ecological, evolutionary & environmental sciences

For a reference copy of the document with all sections, see nature.com/documents/nr-reporting-summary-flat.pdf

Life sciences study design

All studies must disclose on these points even when the disclosure is negative.

Sample size	No statistical test was used to determine sample size. For standard experimental procedures, sample sizes from our previous experience were used (Jones et al., 2019, Nat Comms;). All mouse experiments are performed with more than 3 animals per group. For simpler experiments like food intake or sucralose consumption the number of animals was generellay limited to 4-6. for more complex models, such as tipe 1 diabetes the number of mice was increased to compensate for increased biological variability.
Data exclusions	No data was excluded.
Replication	All experiments were replicated as described in the figure legend.
Randomization	All animals used were aged 6 weeks or older and litter mates were randomly assigned to experimental groups. for studies involving body weight and body composition, body weight was measured at the beginning of the experiments and randomized to avoid significant difference in body weight among the different experimental groups. For human experiments no randomization was necessary as isolated T cells were treated with either control media or media with sweetener.<br><br>All metabolic data are assigned a random order before being injected through the LC-MS column.<br>For other experiments (eg. Western blot, differentiation and proliferation assays) no randomization was possible.
Blinding	The investigator organizing the experimental groups and involved in sample collection was not blinded; however, colleagues aiding in data collection were blinded. For in vitro experiments, the investigators were not blinded for group allocation as the same investigator both planned and performed the experiment. As the same investigators were involved in planning, processing, and acquiring the samples, the experiments could not be performed blinded.

Reporting for specific materials, systems and methods

We require information from authors about some types of materials, experimental systems and methods used in many studies. Here, indicate whether each material, system or method listed is relevant to your study. If you are not sure if a list item applies to your research, read the appropriate section before selecting a response.

Materials & experimental systems

n/a	Involved in the study	
☐	☒	Antibodies
☐	☒	Eukaryotic cell lines
☒	☐	Palaeontology and archaeology
☐	☒	Animals and other organisms
☐	☒	Human research participants
☒	☐	Clinical data
☒	☐	Dual use research of concern

Methods

n/a	Involved in the study	
☒	☐	ChIP-seq
☐	☒	Flow cytometry
☒	☐	MRI-based neuroimaging

Antibodies

Antibodies used	Cell Signalling

Antibodies used

Phospho-PLC gamma 1 Tyr783 (CST, 14008; clone D6M9S; Lot 4; 1:1000)
Phospho-ZAP70 Tyr319 (CST,2717, clone 65e4; lot:15; 1:500
Phospho-Lck Tyr505 (CST; 2751; Lot 1; 1:1000)
Phospho-LAT Tyr220 (CST; 3584; Lot 1; 1:1000)
Phospho-STAT5(Y694) (CST 4322P; clone D47E7; lot 4; 1:1000)
Phospho-ERK1/2 (p44/42 MAPK) (CST; 9101; Lot 30; 1:1000)
CD3 zeta (CST 88083, Lot;1. 1:1000)
Lck (CST; 2984, Clone CD88; Lot:1, 1:1000)
ZAP70 (CST; 2705, clone 99F2; lot:10, 1:1000)
Anti-rabbit IgG HRP-Linked (CST 7074 Lot 25; 1:5000)
NaK ATPase (CST; 3010S, lot:5. 1:1000)
Tom20 (CST; 42406s,D8T4N; lot 1; 1:1000)
β-actin (CST 4970; clone I3E5; lot 1:1000)

Abcam
β-actin (Abcam ab8229; lot gr315283-22; 1:1000)
Alexa488 Goat pAb to Ms IgG (Abcam ab150113; GR3353661-2; 1:1000)

LiCOR
IRDye®800CW Donkey anti-mouse (926-32212, Lot C9110232-09, 1:20000)
IRDye®680CW Donkey anti-mouse (926-68022, Lot D00128-03, 1:20000)
IRDye®680LT Donkey anti-Rabbit (926-68023, Lot D10209-15, 1:20000)
RDye®800CW Donkey anti-Rabbit (926-32213, Lot C90806-09, 1:20000)

Invitrogen (eBioscience)
FITC IL-1b-pro (eBioscience; 11-7114-82 clone NJTEN3; lot 234982; 1:250)
FITC-CD4 (eBioscience; 11-0042-85; Clone RM4-5; 2057560; 1:300)
FITC MHC Class II (I-A/I-E) (clone M5/114.15.2; 11-5321-82)
PE-B220 (eBioscience; 12-0452-83; clone RA3-6B2; Lot 4290694; 1:300)
PE-CD44 (eBiosceince; 120441-81; clone IM7; Lot 3389547; 1:300)
PE-Cy7-CD4 (eBioscience; 25-0041-82; clone GK1.5; Lot 1993630; 1:300)
PE-Cy7-Granzyme B (eBioscience; 25-8898-80; clone NGZB; Lot 1986381; 1:250)
PE-Relm alpha 1 (eBioscience; 12-5441-82; clone DS8RELM; Lot 2356244; 1:300)
PE-FOXP3 (eBioscience; 12-5773-82; clone FJK-16s; Lot 2144983; 1:250)
APC-CD25 (eBiocience; 11-0251-82; clone PC61.5; lot 2018381; 1:300)
APC-NK1.1 (eBioscience; 17-5941-82; clone PK136; lot 1998276; 1:300)
APC-780-CD11b (eBioscience; 47-0112-82; clone M1-70; lot 4290718; 1:300)
eFluor450-TCRb (eBioscience; 48-5961-82; clone H57-597; lot 4335198; 1:300)
eFluor450-CD44 (eBioscience; 48-0441-82; clone IM7; lot 1983649; 1:300)
Biotin-anti-CD3e (eBioscience; 130031-85; clone 145-2C11;)
eFluor450-CD8a (eBioscience; 48-0081-82; clone 53-6.7; lot 2093800; 1:300)
eFluor450-CD11c (eBioscience; 48-0114-80; clone N418; lot 1994180)

anti-Mouse CD28 functional grade (clone 37.51; 16-0281-86; 1:500)
anti-Mouse CD3e functional grade (clone 145-2C11; 16-0031-86; 1:200)

TONBO Bioscience
FITC-IFNg (TONBO; 35-7311; clone XMG1.2; C7311092719353; 1:300)
Violet Fluor450-CD4 (TONBO; 750042; clone RM4-5; C0042081618753; 1:300)
PerCP-cyanine5.5-CD4 (TONBO; 65-0041-u100; clone CK1.5; C0041082619653; 1:300)

BioLegend
FITC-Annexin
FITC-F4/80 (Biolegend; 123107; clone BM8; LOT B287205; 1:300)
PE-CD4 (Biolegend; 100408; clone GK1.5; LOT B248731; 1:300)
PE-CD8a (Biolegend; 100708; clone 53-6.7; LOT B268831; 1:300)
PE XCR1 (Biolegend; 148203; clone ZET; LOT B)
PE-TCR beta chain (Biolegend; 109207; clone H57-597; LOT B308457)
PerCP-Cy5.5 B220 (clone RA3-6B2; 103236)
PerCP-Cy5.5 Ki67 (Biolegend; 652424; clone16A8; lot b235166; 1:250)
PE-Cy7-Tbet (Biolegend; 644824; clone 4B10; lot b255602; 1:250).
PE-Cy7-CD206 (Biolegend; 141719; clone C068C2; lot b318357; 1:300)
APC-TNF (Biolegend; 506308; clone MP6-XT22; lot b255367; 1:250)
APC-GL7 (Biolegend; 144618; clone GL7; lot b287646; 1:300)
APC-CD45RB (Biolegend; 103319; clone C363-16A; lot 2082880; 1:300)
APC-CD301 (Biolegend; 145707; clone LOM-14; lot b312436; 1:300)
Alexa Fluor 647 Sirp alpha (Biolegend; 144028; clone p84; lot ; 1:300)
BV421-Tbet (Biolegend; 644815; clone 4B10; lot b317403; 1:250)
BV605-CD95 (Biolegend; 152612; clone SA367H8; lot b261359; 1:300)
BV650-CD69 (Biolegend; 104541); clone H1.2F3; lot b258598; 1:300)
BV650 CD11c (Biolegend; 117339; clone N418; lot)
Anti-mouse IL4 (clone 11B11; 504102)
FITC-CD8a (Biolegend; 100706; clone53-6.7; lot b298556; 1:300)
BV711-CD4 (Biolegend; 100557; clone RM4-5; lot b35831; 1:300)
FITC-NK1.1 (Biolegend; 108706; clone PK136; lot b236605; 1:300)
BV785-B220 (Biolegend; 103246; clone RA3-6B2; lot b256836; 1:300)

PeCy7-CD8 alpha (Biolegend; 100722; clone 53-6.7; lot b357207; 1:300)

BD-Pharmigen
PE-Cy7-CD45.1 (BD; 560578; clone A20; LOT 0307154; 1:300)
PE-CD184 (BD; 551955; Lot 1204181; 1:300)

Baylor College of Medicine - MHC Tetramer Facility
PE-Kb/OVA257 (19027)

Santa Cruz (SC)
PLCg1 (SC; sc7290; clone E-12, lot: H1518; 1:1000)
STAT5 (SC; sc74442; clone A-9; LOT F1421; 1:1000)
GAPDH (SC; sc-322333, Clone D0722, lot: ; 1:1000)
ZAP70 (SC; sc-32760; clone 1E7.2; LOT D1217; 1:1000)
Lck (SC; sc-433; clone 3A5; LOT C2519; 1:1000)
LAT (SC; sc-53550; clone 11b.12; LOT C1722; 1:500)
ERK1/2 (SC; sc-514302; clone C9; LOT C1722; 1:1000)
CD3 zeta (clone 6B10.2; sc-1239)
Mouse IgG (sc-2025, Lot2721, for IP)

Invitrogen Thermo Fisher Scientific
Alexa Fluor 546 goat anti-hamster IgG (Invitrogen; A21111; lot 2349086; 1:1000)

Validation

All antibodies are commercially available. Antibodies employed here in our manuscript were previously reported and routinely used for the application used. All companies used report quality control measures to ensure reproducibility. CST adheres to the Hallmarks of Antibody Validation™, six complementary strategies that can be used to determine the functionality, specificity, and sensitivity of an antibody in any given assay, which is adapted from Uhlen, et. al., ("A Proposal for Validation of Antibodies." Nature Methods (2016). Abcam's validation is application-specific to be effective and information on which applications an antibody has been validated in can be found in the Tested Applications section on any antibody datasheet.

Eukaryotic cell lines

Policy information about cell lines

Cell line source(s)

EG7OVA (EL4OVA); KC (pdx1-KrasG12D); Jurkat (cloneE6-1)

Authentication

pdx1-KrasG12D cell line was isolated from primary pancreatic tumours from a mouse model of pancreatic cancer and their background purity where authenticated by SNP analysis. The Eg7ova cell line was authenticated by PCR analysis. Jurkat (cloneE6-1) was validated by the Francis Crick cell service using STR (Short Tandem Repeat).

Mycoplasma contamination

EG7OVA, KC (pdx1-KrasG12D) and Jurkat (cloneE6-1) tested negative for mycoplasma contamination.

Commonly misidentified lines
(See ICLAC register)

No commonly misidentified cell lines were used in the study.

Animals and other organisms

Policy information about studies involving animals; ARRIVE guidelines recommended for reporting animal research

Laboratory animals

All laboratory animals used were of pure C57/B6J, FVB, or NOD background (as indicated in the figures), female-male mice ages 6-12 weeks. Mice used for the LPS-induced sepsis model were females aged 8-10weeks. Mice used for the EL4 OVA experiments were older than 6-10 weeks. Female NODShlTj mice were purchased at 7 weeks of age. Male FVB (>10 weeks of age) were used for the MHC-mismatch experiments. Rag2-/- OTI mice used were male aged 6-10 weeks. OT I cells were adoptively transferred into C57Bl/9J male recipients aged 6-9 weeks. Rag2-/- mice used in the study were male aged 6-10 weeks of age. TCRα -/- mice were male aged 6-8 weeks. B6.SJL-PtprcaPepcb/BoyCrl congenic mice were male aged 6-10 weeks. Mice allocated for bone marrow derived macrophages and T cells were mixed sex (male or female) with ages ranging from 8-14 weeks.

Wild animals

There were no wild animals used in this study.

Field-collected samples

No field-collected samples were used in this study.

Ethics oversight

Animal experiments were subject to ethical review by the Francis Crick Animal Welfare and Ethical Review Body and regulation by the UK Home Office project licence P319AE968. All mice were housed under conditions in line with the Home Office guidelines (UK). Metabolic animal studies were approved by the Animal Ethics Committee of the government of Upper Bavaria (Germany). All procedures were performed following the Animals (scientific procedures) Act 1986 and/or the EU Directive 2010.

Note that full information on the approval of the study protocol must also be provided in the manuscript.

Human research participants

Policy information about studies involving human research participants

Population characteristics

The study was performed on a population of healthy adults aged 18 - 70 years old and included men and women. Participants

Population characteristics | were excluded if they had an immune-mediated disease, cancer in the past 5 years or had current/recent symptoms of viral or other infection. Participants using medication, such as statins, with immune response modifying effects, were also excluded. All samples were collected between 0800 and 1200

Recruitment | Participants were recruited from the staff and student populations at Swansea University, Wales UK. Potential participants responded to ethics committee approved advertising by contacting the local clinical research facility. The clinical research facility oversaw recruitment through informed written consent in response to an ethically approved participant information sheet that explained the study. Participant recruitment was conducted by the Joint Clinical Research Facility at Swansea University with no selection bias.

Ethics oversight | This project was approved by Wales Research Ethics Committee 6 (approval 13/WA/0190) which is a committee within the Health Research Authority structure within the UK and equivalent to Institutional Review Board in USA. Peripheral blood was collected from healthy, non-fasted individuals. Informed written consent and ethical approval was obtained from Wales Research Ethics Committee 6 (13/WA/0190).

Note that full information on the approval of the study protocol must also be provided in the manuscript.

Flow Cytometry

Plots

Confirm that:

☒ The axis labels state the marker and fluorochrome used (e.g. CD4-FITC).

☒ The axis scales are clearly visible. Include numbers along axes only for bottom left plot of group (a 'group' is an analysis of identical markers).

☒ All plots are contour plots with outliers or pseudocolor plots.

☒ A numerical value for number of cells or percentage (with statistics) is provided.

Methodology

Sample preparation | Sample preparation of biological sources (human blood, murine bone marrow) is as described in the methods section of the manuscript . Single cell suspensions from tumours, spleen, and lymph nodes were prepared as described in the methods section. T cells were isolated and prepared for flow cytometry as described in methods. Macrophages were differentiated, stimulated, and prepared as written in the methods section.

Instrument | BD Symphony; BD LSRFortessa .

Software | BD FACSDIVA V 8.0.1

Cell population abundance | Post sorting CD4+ CD45RB+ cells for the T cell colitis transfer model was >90%.

Gating strategy | Live cells were gated using viability exclusion dyes. Lymphocyte populations were identified using FSC-H and SSC-A. Single cells were identified using the FSC-H v FSC-A gates. Lymphocytes and macrophages were stained with a target of interest whereby gating was determined using an unstained vs stained sample.

☒ Tick this box to confirm that a figure exemplifying the gating strategy is provided in the Supplementary Information.
