# Supplementary material for: The dietary sweetener sucralose is a negative modulator of T cell-mediated responses
Source: Nature. 2023 Mar 15;615(7953):705–11. doi: 10.1038/s41586-023-05801-6 (PMC10033444; doi:10.1038/s41586-023-05801-6)

The dietary sweetener sucralose is a negative modulator of T-cell-mediated responses

Fabio Zani*, Julianna Blagih*, Tim Gruber, Michael D. Buck, Nicholas Jones, Marc Hennequart, Clare L. Newell, Steven E. Pilley, Pablo Soro-Barrio, Gavin Kelly, Nathalie M. Legrave, Eric C. Cheung, Ian S. Gilmore, Alex P. Gould, Cristina Garcia-Caceres, Karen H. Vousden

Supplementary Information

Raw Gels for Western Blots

Raw Gel for Figure 2b. PLCγ phosphorylation downstream of TCR stimulation in primary murine T cells
Molecular weights indicated to the left of the gel
Lanes (left to right)
1) control unstimulated; 2) control 1 min αCD3; 3) control 2 min αCD3; 4) control 5 min αCD3.
5) sucralose unstimulated; 6) sucralose 1 min αCD3; 7) sucralose 2 min αCD3; 8) sucralose 5 min αCD3.
Phospho-PLCγ (left gel) and total PLCγ (right gel).

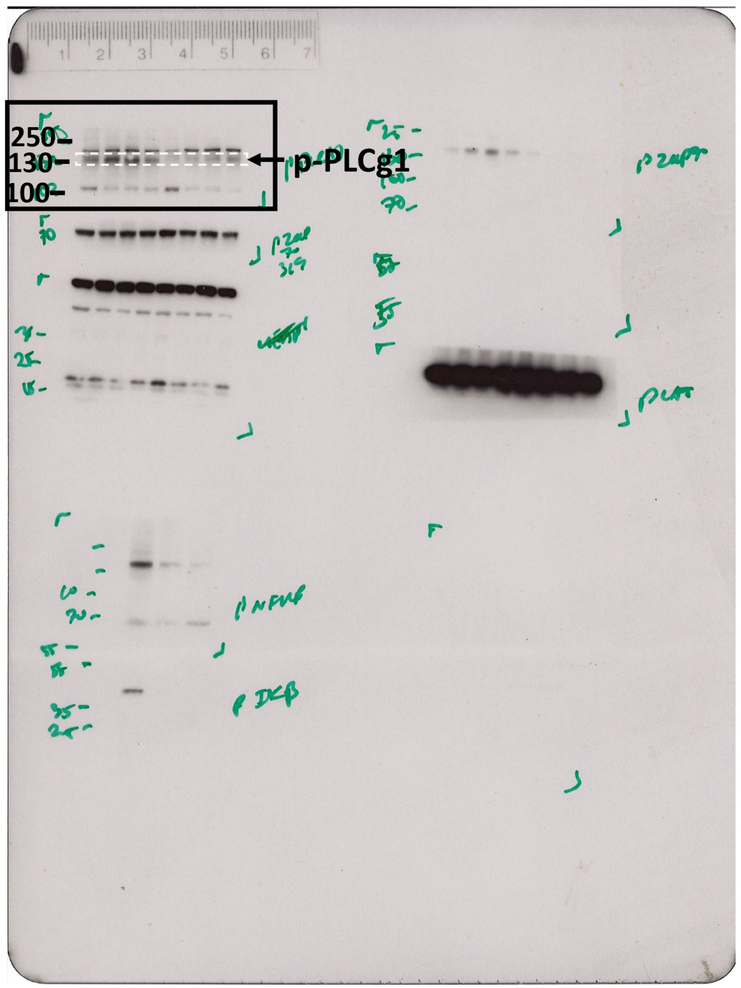

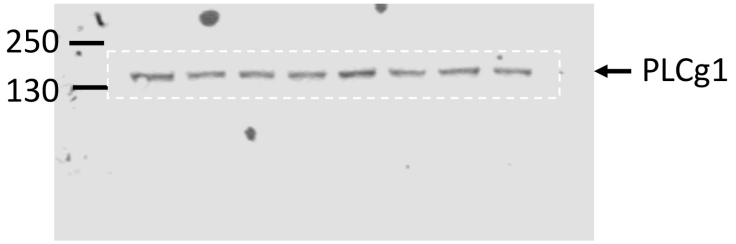

Raw Gel for Extended Data Fig. 5i. STAT5 activity in primary murine T cells activated for 24hours with αCD3/CD28
Molecular weights indicated to the left of the gel
Lanes (left to right)
1) control T cells 2) control T cells 3) control T cells; 4) Sucralose-treated T cells; 5) sucralose-treated T cells; 6) sucralose-treated T cells.
Phospho-STAT5 (left gel) and total STAT5 (right gel).

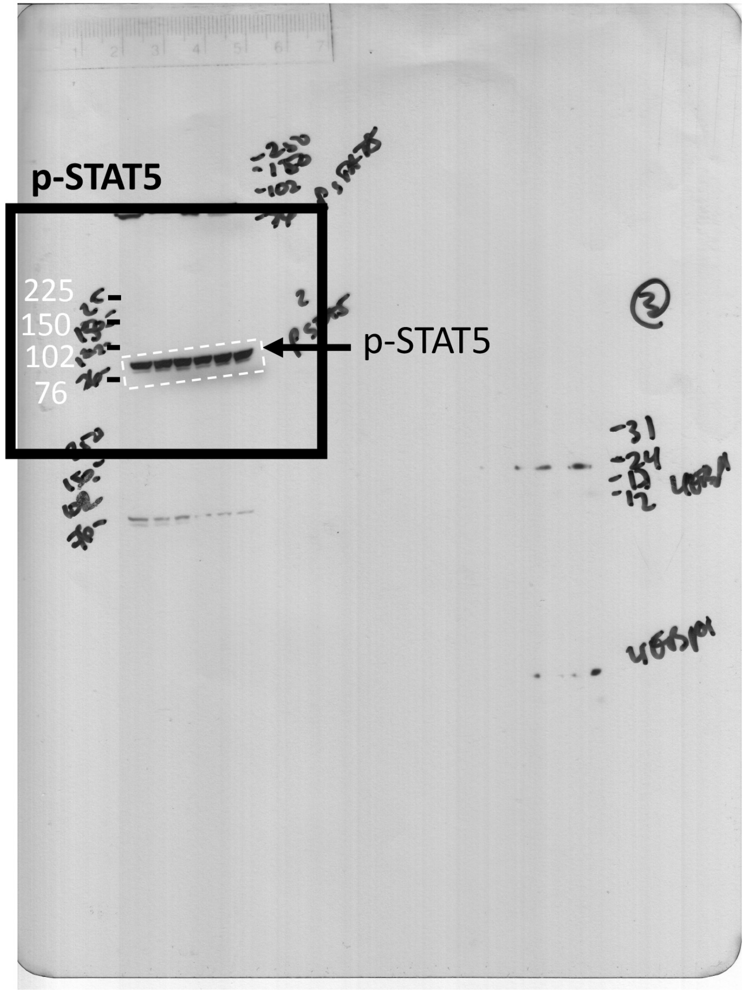

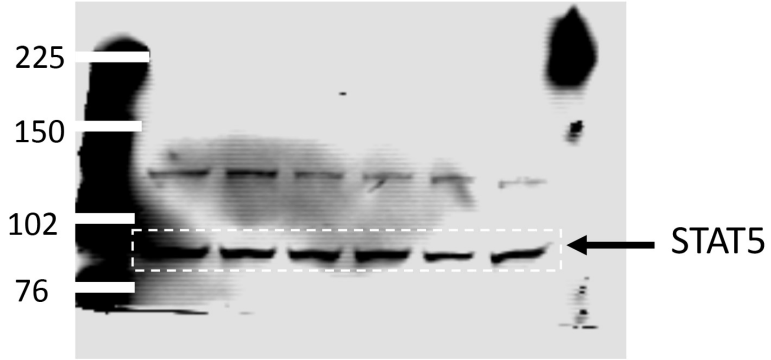

Raw Gel for Extended Data Fig. 6b. PLCγ phosphorylation in Jurkat T cells.
Molecular weights indicated to the left of the gel
Lanes (left to right)
1) Control Jurkat cells; 2) Sucralose-treated Jurkat cells
Phospho-PLCγ (top gel) and total PLCγ (bottom gel).

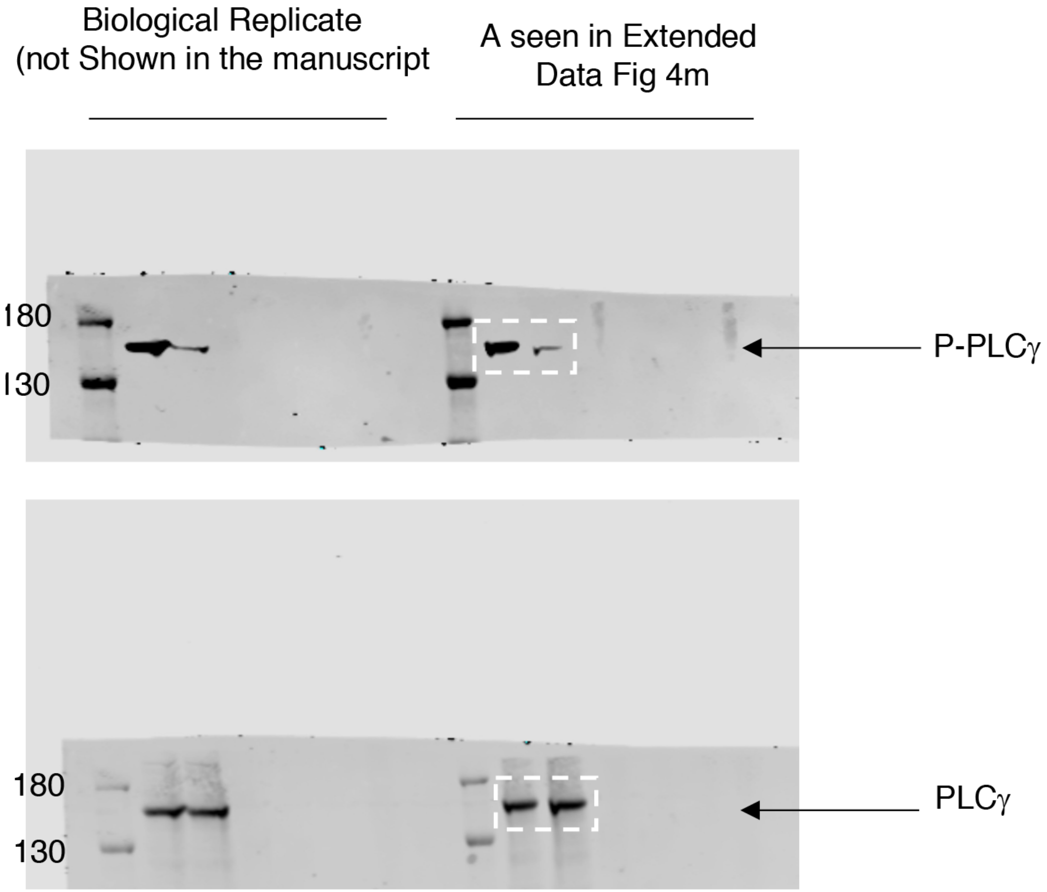

Raw Gel for Extended Data Fig. 6c. ERK activity in primary murine T cells downstream of TCR activation
Molecular weights indicated to the left of the gel
Lanes (left to right)
1) control unstimulated; 2) control 1 min αCD3; 3) control 2 min αCD3; 4) control 5 min αCD3.
5) sucralose 1 min αCD3; 6) sucralose 2 min αCD3; 7) sucralose 5 min αCD3.
Phospho-ERK (left gel), total ERK (right bottom gel), and β-Actin (right top gel).

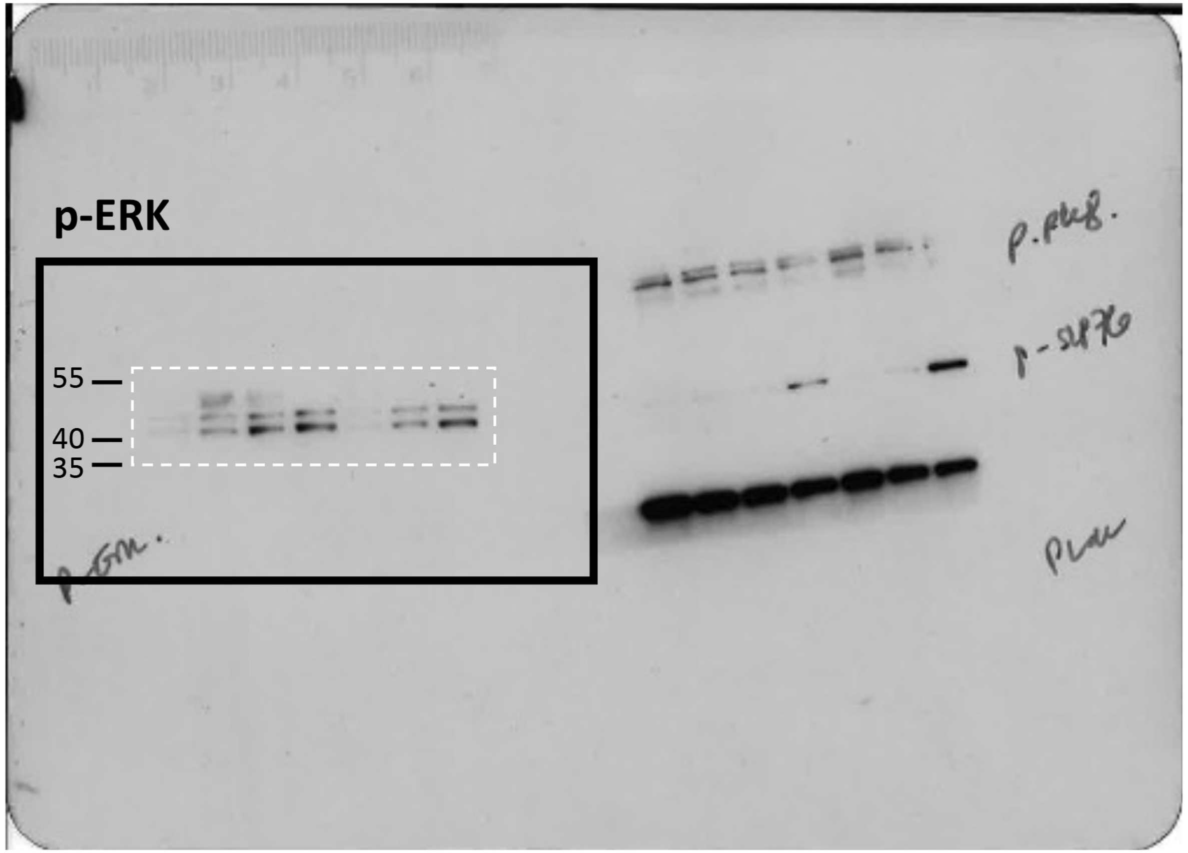

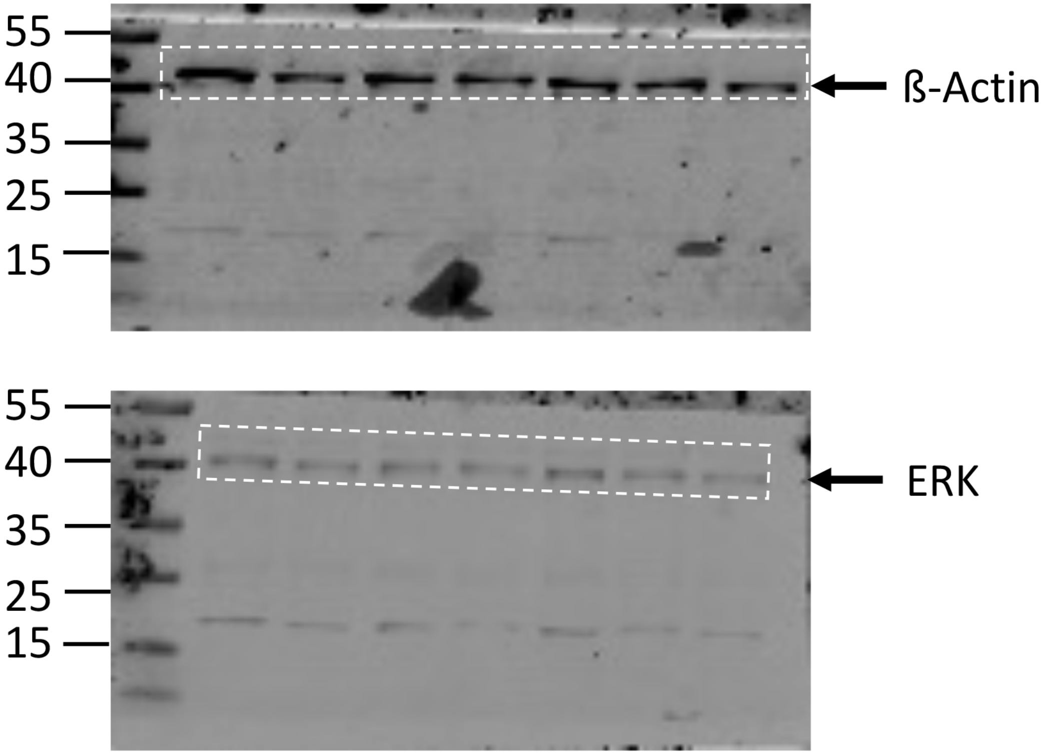

Raw Gel for Extended Data Fig. 6d. ZAP70 activity in primary murine T cells downstream of TCR activation

Molecular weights indicated to the left of the gel

Lanes (left to right)

1) control unstimulated; 2) control 1 min αCD3; 3) control 2 min αCD3; 4) control 5 min αCD3.

5) sucralose 1 min αCD3; 6) sucralose 2 min αCD3; 7) sucralose 5 min αCD3.

Phospho-ZAP70[Tyr319] (left gel), total ZAP70 (right top gel), and β-Actin (right bottom gel).

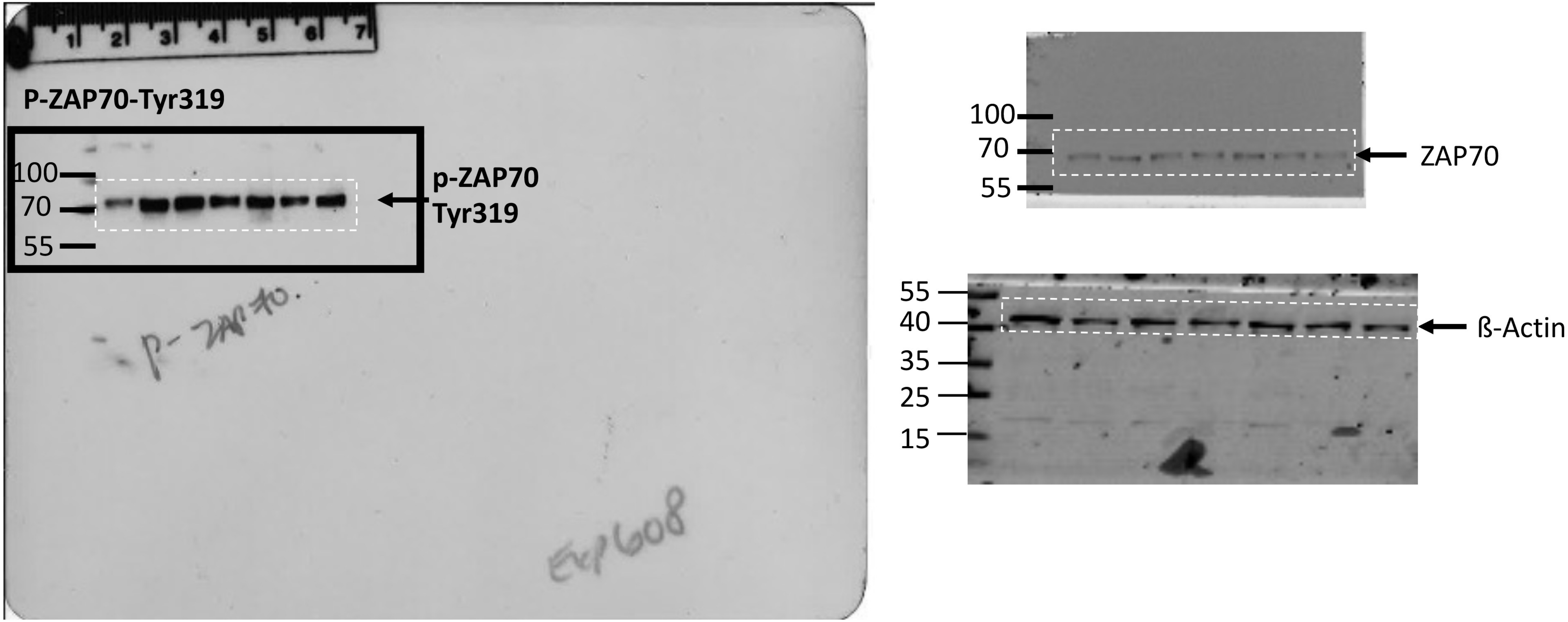

Raw Gel for Extended Data Fig. 6d. Lck activity in primary murine T cells downstream of TCR activation
Molecular weights indicated to the left of the gel
Lanes (left to right)
1) control unstimulated; 2) control 1 min αCD3; 3) control 2 min αCD3; 4) control 5 min αCD3.
5) sucralose 1 min αCD3; 6) sucralose 2 min αCD3; 7) sucralose 5 min αCD3.
Phospho-Lck (left gel), total Lck (right top gel), and Vinculin (right bottom gel).

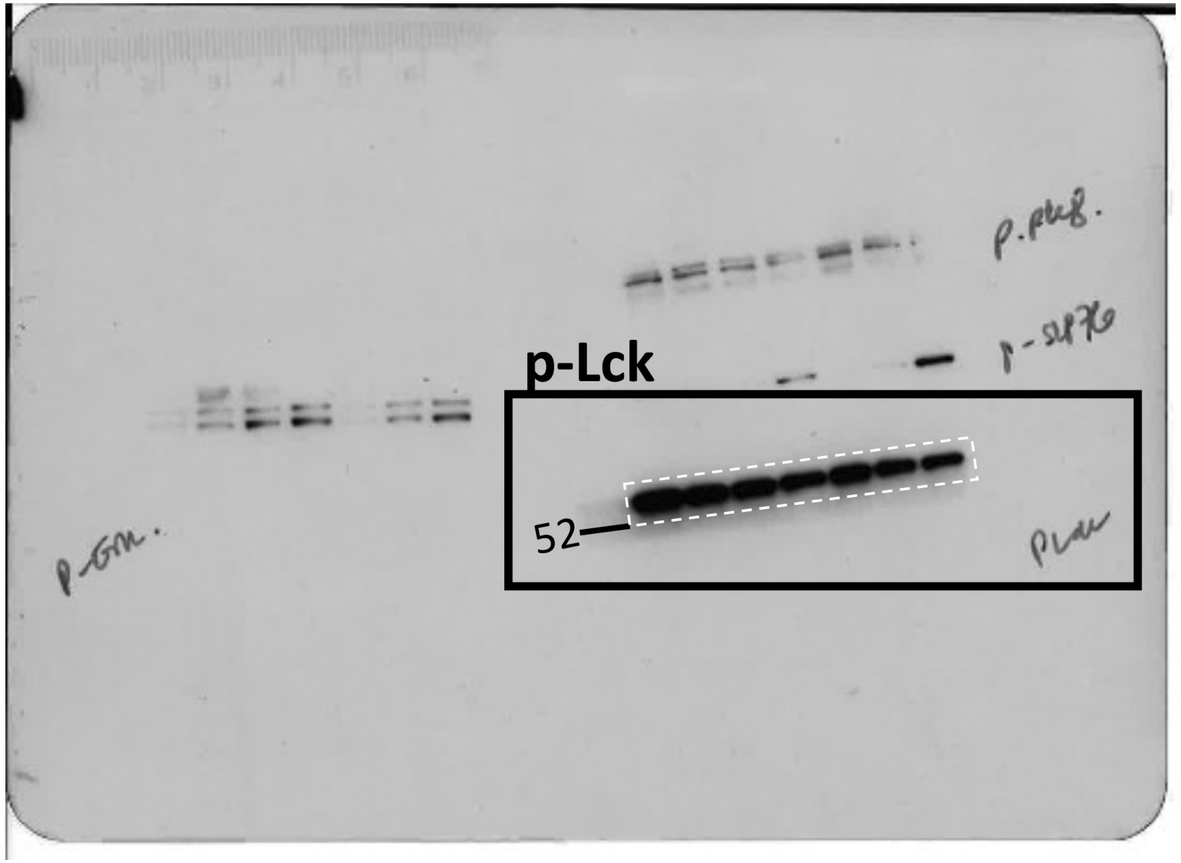

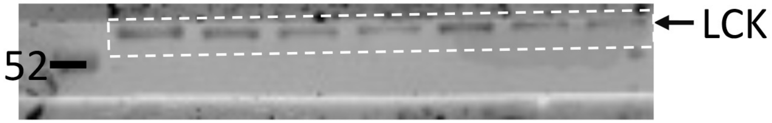

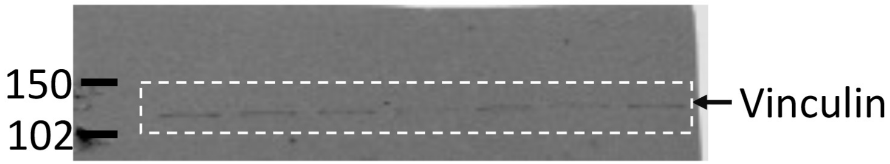

Raw Gel for Extended Data Fig. 6d. LAT activity in primary murine T cells downstream of TCR activation

Molecular weights indicated to the left of the gel

Lanes (left to right)

1) control unstimulated; 2) control 1 min αCD3; 3) control 2 min αCD3; 4) control 5 min αCD3.

5) sucralose 1 min αCD3; 6) sucralose 2 min αCD3; 7) sucralose 5 min αCD3.

Phospho-LAT (left gel), total LAT (right top gel), and β-Actin (right bottom gel).

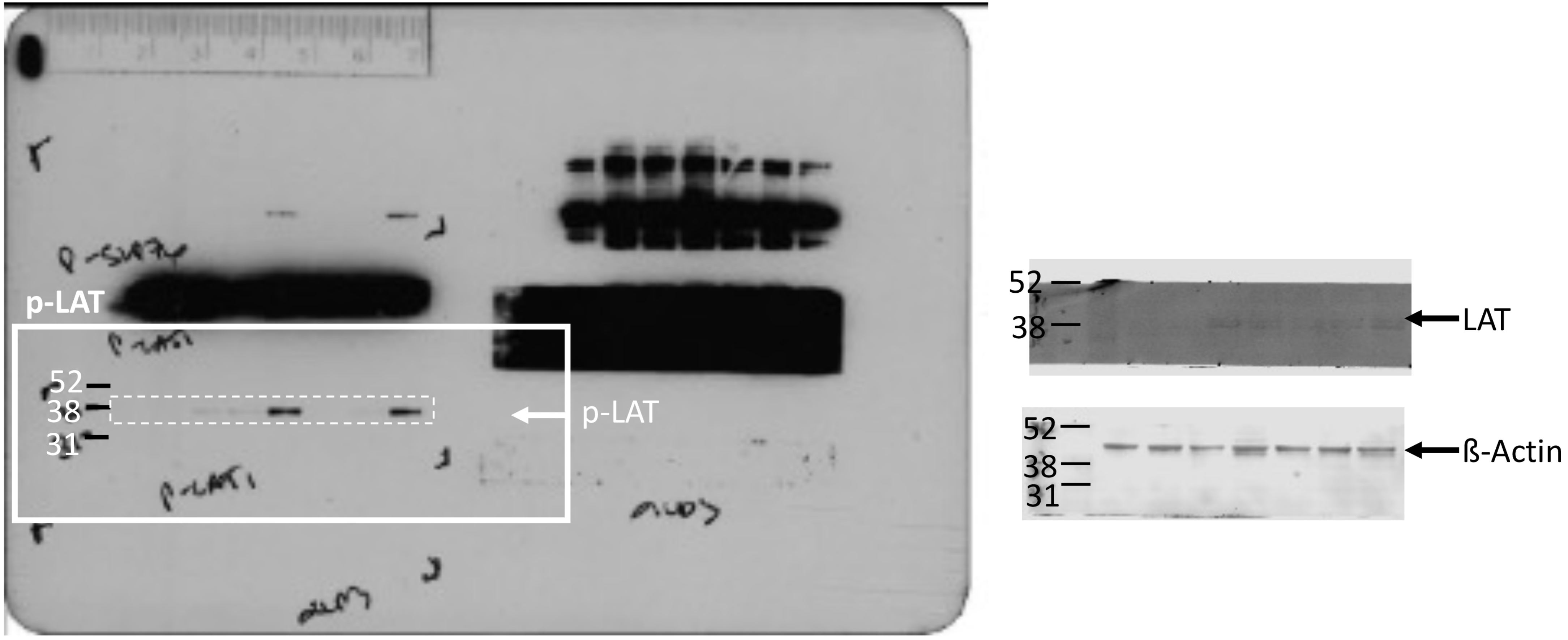

Raw Gel for Extended Data Fig. 6e. Immunoprecipitation for ZAP70 and CD3ζ in Jurkat T cells

Molecular weights indicated to the left of the gel

Lanes (left to right)

1) control unstimulated; 2) control 1 min αCD3; 3) control 2 min αCD3; 4) control 5 min αCD3.

5) sucralose unstimulated; 6) sucralose 2 min αCD3; 7) sucralose 5 min αCD3. for IP only 6) empty 7)and 8) IgG CTR

Left: (top to bottom) ZAP70, Lck, CD3ζ. Right (top to bottom) ZAP70, total Lck, CD3 β-Actin (right bottom gel).

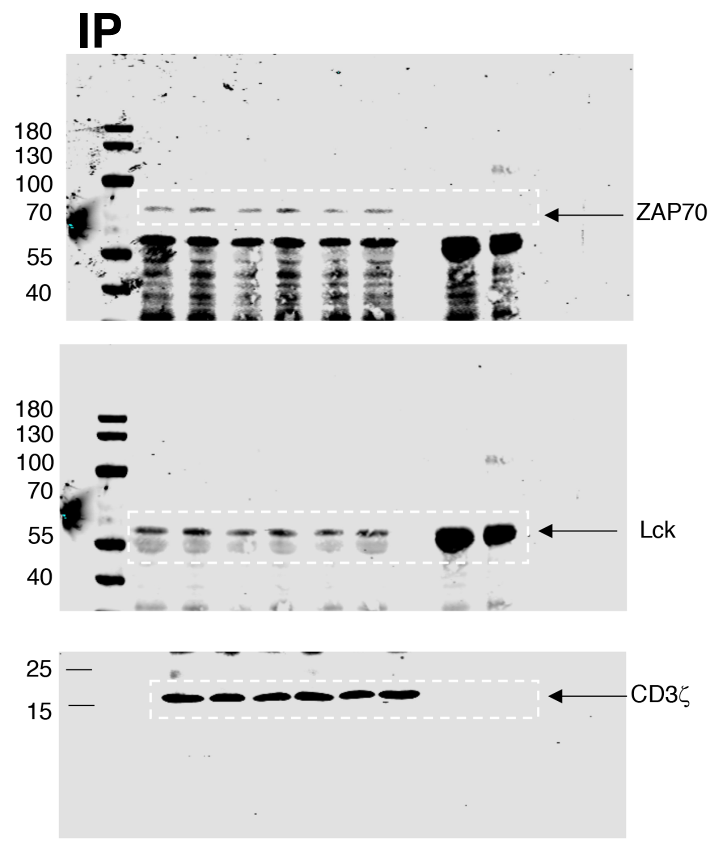

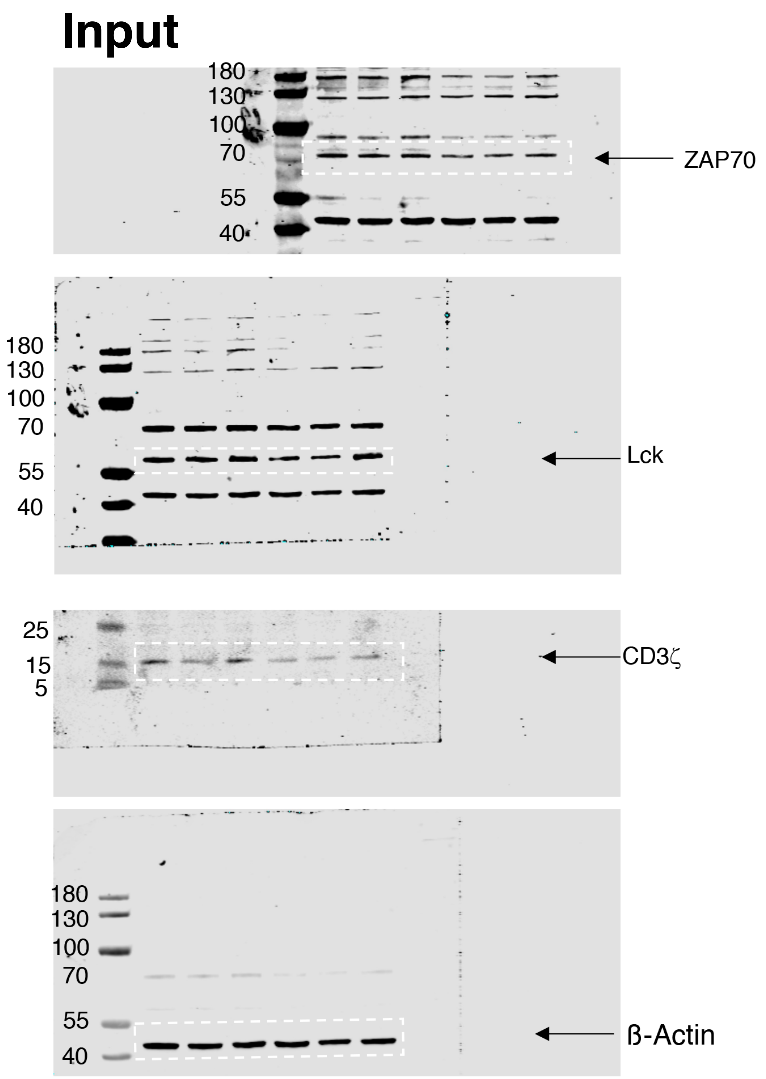

Raw Gel for Extended Data Fig. 6f. Subcellular fractionation of Jurkat T cells.
Molecular weights indicated to the left of the gel
Lanes (left to right)
1) and 2) Whole cell fraction; 3) and 4) Cytoplasmic fraction; 5) and 6) membrane fraction
top to bottom: Na-K ATPase; GAPDH, Tom20

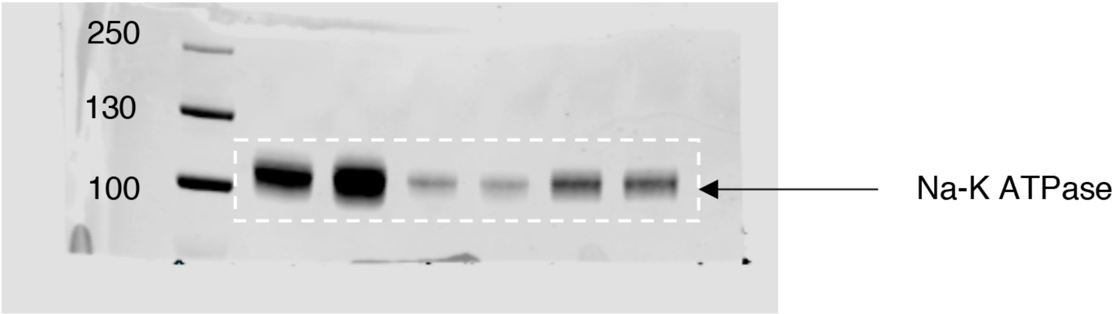

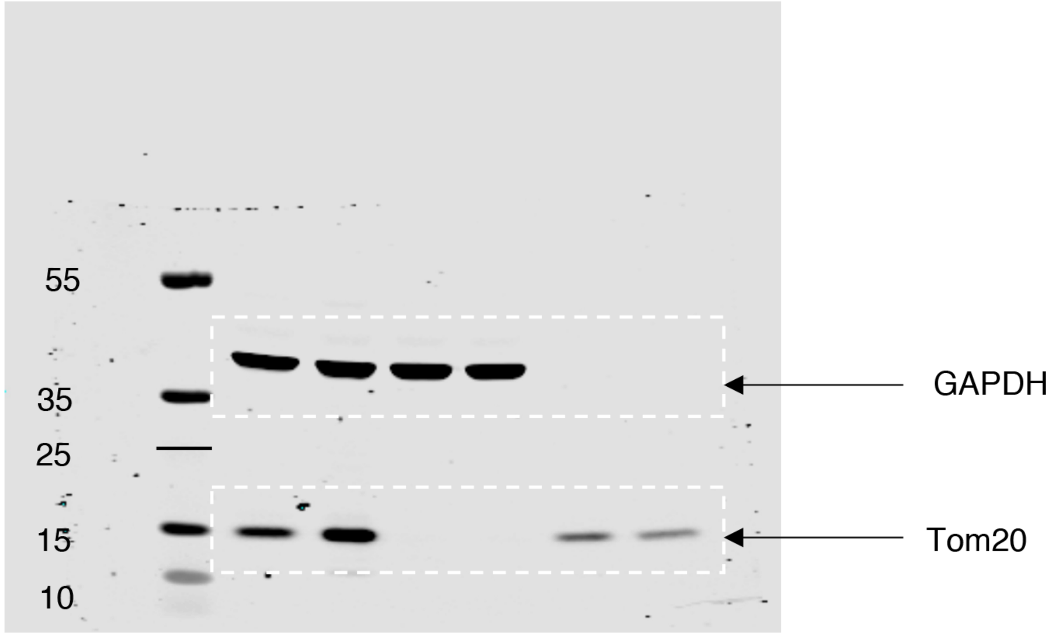

Supplement: Supplementary file 3 — Uncropped western blots [file 41586_2023_5801_MOESM3_ESM.pdf]
